# *Cryptosporidium* Priming Is More Effective than Vaccine for Protection against Cryptosporidiosis in a Murine Protein Malnutrition Model

**DOI:** 10.1371/journal.pntd.0004820

**Published:** 2016-07-28

**Authors:** Luther A. Bartelt, David T. Bolick, Glynis L. Kolling, James K. Roche, Edna I. Zaenker, Ana M. Lara, Francisco Jose Noronha, Carrie A. Cowardin, John H. Moore, Jerrold R. Turner, Cirle A. Warren, Gregory A. Buck, Richard L. Guerrant

**Affiliations:** 1 Division of Infectious Diseases, University of North Carolina at Chapel Hill, Chapel Hill, North Carolina, United States of America; 2 Division of Infectious Diseases and Center for Global Health, University of Virginia, Charlottesville, Virginia, United States of America; 3 Molecular Biology and Genetics, Virginia Commonwealth University, Richmond, Virginia, United States of America; 4 Department of Pathology, The University of Chicago, Chicago, Illinois, United States of America; 5 Departments of Pathology and Medicine—Gastroenterology, Brigham and Women’s Hospital, Boston, Massachusetts, United States of America; University of California San Diego School of Medicine, UNITED STATES

## Abstract

*Cryptosporidium* is a major cause of severe diarrhea, especially in malnourished children. Using a murine model of *C*. *parvum* oocyst challenge that recapitulates clinical features of severe cryptosporidiosis during malnutrition, we interrogated the effect of protein malnutrition (PM) on primary and secondary responses to *C*. *parvum* challenge, and tested the differential ability of mucosal priming strategies to overcome the PM-induced susceptibility. We determined that while PM fundamentally alters systemic and mucosal primary immune responses to *Cryptosporidium*, priming with *C*. *parvum* (10^6^ oocysts) provides robust protective immunity against re-challenge despite ongoing PM. *C*. *parvum* priming restores mucosal Th1-type effectors (CD3^+^CD8^+^CD103^+^ T-cells) and cytokines (IFNγ, and IL12p40) that otherwise decrease with ongoing PM. Vaccination strategies with *Cryptosporidium* antigens expressed in the *S*. Typhi vector 908*htr*, however, do not enhance Th1-type responses to *C*. *parvum* challenge during PM, even though vaccination strongly boosts immunity in challenged fully nourished hosts. Remote non-specific exposures to the attenuated *S*. Typhi vector alone or the TLR9 agonist CpG ODN-1668 can partially attenuate *C*. *parvum* severity during PM, but neither as effectively as viable *C*. *parvum* priming. We conclude that although PM interferes with basal and vaccine-boosted immune responses to *C*. *parvum*, sustained reductions in disease severity are possible through mucosal activators of host defenses, and specifically *C*. *parvum* priming can elicit impressively robust Th1-type protective immunity despite ongoing protein malnutrition. These findings add insight into potential correlates of *Cryptosporidium* immunity and future vaccine strategies in malnourished children.

## Author Summary

*Cryptosporidium* attributable morbidities in malnourished children are increasingly recognized. Exactly how malnutrition interferes with host mucosal immunity to diarrheal pathogens and mucosal vaccine responses remains unclear. Dissecting these interactions in an experimental model of cryptosporidiosis can uncover new insights into novel therapeutic approaches against a pathogen for which effective therapies and vaccines are currently unavailable. We demonstrate that although malnutrition diminishes baseline (primary) Th1-type mucosal immunity these deficits can be partially overcome via non-specific mucosal strategies (*S*. Typhi and CpG) and completely restored after a sub-clinical (low-dose) exposure to viable *C*. *parvum*. These results add insight into preventive strategies to help alleviate *Cryptosporidium*-specific diarrhea in children in low-resource settings and abrogate prolonged post-infection sequelae.

## Introduction

Malnutrition affects an estimated 165 million children worldwide [1], contributes to an estimated 45% of early childhood mortality [2], and interferes with immune responses to enteric pathogens and mucosally delivered vaccines [3]. *Cryptosporidium sp*., a ubiquitous waterborne apicomplexan intestinal protozoan, is a prototypic pathogen that is more severe in malnourished children. Independent of socioeconomic status, early childhood *Cryptosporidium* infection associates with excess mortality in West Africa (hazard ratio 2.9; 1.7–4.9), sub-Saharan Africa, and South Asia (HR 2.3; 1.3–4.3) where malnutrition prevalence remains high. *Cryptosporidium* infection associates with up to a 4-fold risk for persistent diarrhea (>14 days) [4–7] increases likelihood of recurrent diarrheal episodes, and associates with growth decrements [8, 9]. Even non-diarrheal *Cryptosporidium* infections can acutely impair growth [10], and sustained linear growth shortfalls may persist for months following infection [11, 12].

While severe manifestations of *Cryptosporidium* infection in patients living with advanced HIV/AIDS [13] and studies in animal models confirm an undisputed role for Th1-effector mediated clearance of *Cryptosporidium* [14–16], whether and how malnutrition increases susceptibility to *Cryptosporidium* in children is not well understood. Unlike the protective effect of IFN-γ seen in jejunal tissues of sensitized healthy volunteers who rapidly clear *Cryptosporidium* [17], fecal IFN-γ levels are paradoxically lower in malnourished children infected with *Cryptosporidium* than uninfected controls [18, 19]. In contrast, stool cytokines in malnourished children with active cryptosporidiosis demonstrate increased TNF-α, IL-8, and IL-13, and serum IgE, but not IgG is elevated [19]. Also, whereas circulating CD4^+^ and CD8^+^ T-cells from infected individuals produce IFN-γ upon re-stimulation with *Cryptosporidium* antigens [20], cell-mediated immune (CMI) responses are generally impaired during infection in malnourished children, but serum and fecal antibodies are increased [21]. Whether this apparent skew in the immune response is characteristic not only of active, but also responses to recurrent *Cryptosporidium* infection in malnourished children has yet to be determined. Although reduced systemic IFN-γ has been documented in some malnourished children [2], CD4^+^ T-cell quantity and activation is not consistently impaired, and one follow-up study in malnourished children demonstrated partial reconstitution of CMI through six weeks post-*Cryptosporidium* infection [21].

We have previously reported that undernourished neonatal and weaned mice have enhanced susceptibility to *Cryptosporidium* [22–24] infection concurrent with diminished baseline mucosal IFN-γ secretion [23]. Restoration of IFN-γ levels via systemic exposure to the TLR9 agonist CpG immediately prior to infection can partially attenuate *Cryptosporidium* susceptibility during malnutrition [24]. Despite apparently diminished basal IFN-γ responses during malnutrition, however, mice vaccinated with the *Salmonella enterica* serovar Typhi strain CVD 908-htr intranasal vector expressing the *Cryptosporidium* sporozoite antigen Cp15 [25] had unexpectedly preserved splenocyte CMI, including IFN-γ recall responses, through two weeks post vaccination [26]. This finding coupled with a rise in IFN-γ at later timepoints post-infection [27] suggests that despite constitutively diminished IFN-γ secretion, these malnourished hosts could produce IFN-γ in adaptive responses.

In the present study, we dissected how protein malnutrition influences both primary and secondary immune responses to natural *C*. *parvum* infection and tested whether mucosal delivered strategies could overcome the resultant immunodeficiency. We first established that severe protein malnutrition (PM) differentially recapitulates short and long-term features of severe childhood cryptosporidiosis, and combines with *C*. *parvum* to worsen intestinal epithelial cell tight-junction disruption and mucosal architecture. These changes were co-incident with a fundamentally altered basal (or primary) immune response to *Cryptosporidium* antigens in both the systemic and mucosal compartment that resembled findings in malnourished children (i.e. increased IL13, decreased IFNγ). Surprisingly, however, Th1-type secondary responses were not only preserved, but enhanced during PM. Even at low (10^6^) doses in this model, priming with viable *C*. *parvum* oocysts was sufficient to provide protective immunity. CD8^+^ T-cells predominated the secondary mucosal immune response, and were accompanied by Th-1 type cytokines (IFNγ, IL12p40) along with the lymphocyte chemoattractant, CCL5. Vaccination with either of two *Cryptosporidium* sporozoite antigens expressed in the *S*. Typhi vector, however, was unable to provide protective immunity in malnourished mice, despite robust boosted responses in nourished hosts. We also found that while remote non-specific mucosal exposures to either *S*. Typhi or CpG could partially attenuate the course of cryptosporidiosis during PM, neither alone nor in combination was as effective as *C*. *parvum* priming.

## Results

### Protein malnutrition differentially enhances gut disruption and weight loss during *C*. *parvum* infection in mice

**Fig 1:**
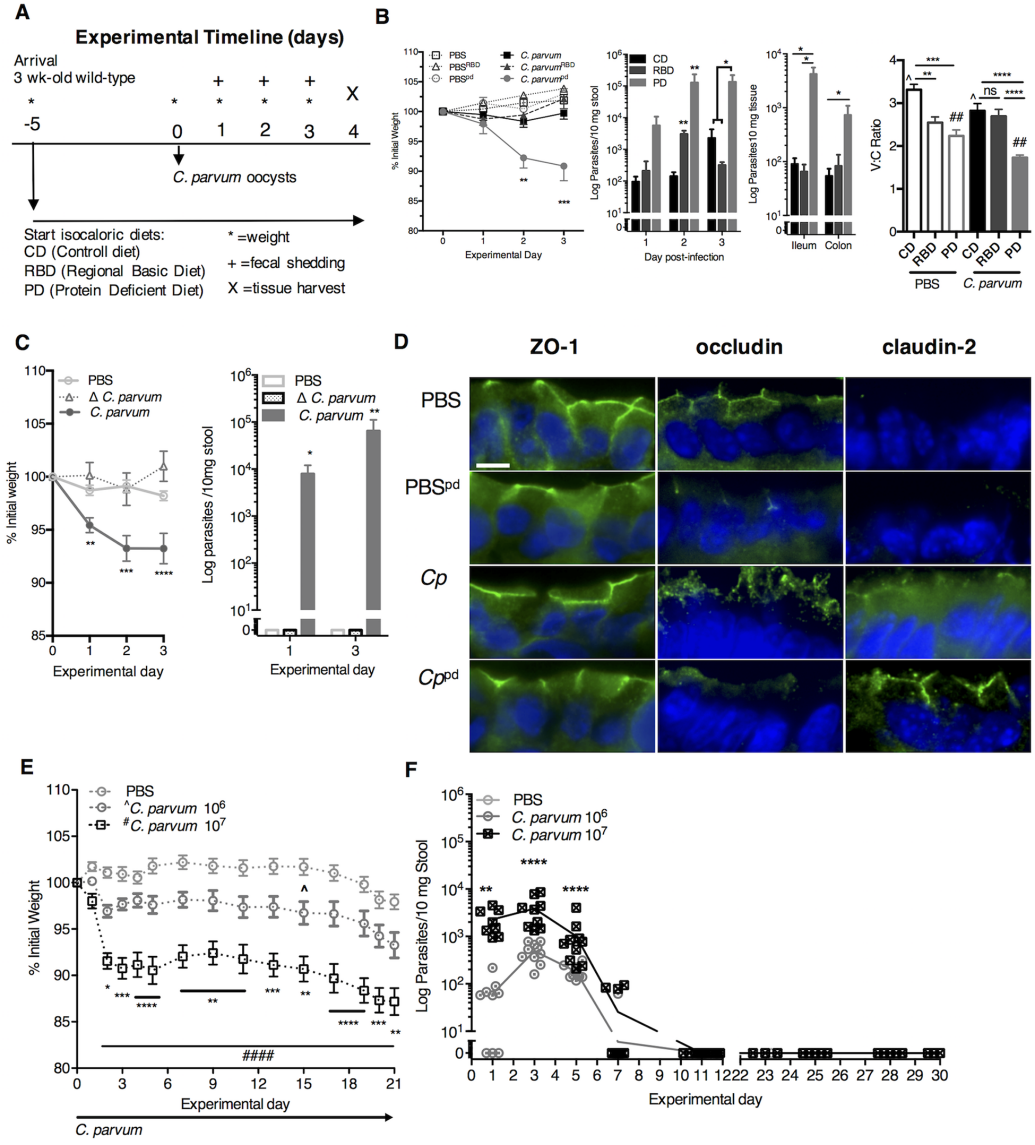
Severe protein malnutrition in mice selectively enhances intestinal disruption and severity of cryptosporidiosis. (A) Experimental timeline. 3-week-old C57Bl/6 female mice were initiated on experimental malnutrition diets (RBD or PD) or control diet (CD) immediately upon receipt from supplier. Challenge with 10^6^ or 10^7^ *Cryptosporidium parvum* oocysts, heat-inactivated *C*. *parvum* oocysts (Δ*C*. *parvum*) or PBS occurred via oral gavage 5 days after initiating diet. Serial weights were collected daily post-challenge, and fecal parasite shedding was determined by RT-PCR. On day 3–4 post-challenge, tissue parasite burden and mucosal injury was assessed by measuring ileum villus:crypt ratios and alterations in epithelial tight-junction proteins. (B) Impact of diet on growth (**P<0.01, ***P<0.001 *C*. *parvum*^PD^ vs. *C*. *parvum* or *C*. *parvum*^RBD^), fecal parasite shedding (**P<0.01 PD or RBD vs. CD day 2, *P<0.05 PD vs. RBD or CD day 3), tissue parasite burden (*P<0.05 PD vs CD or RBD ileum, *P<0.05 PD vs CD colon), and ileum villus:crypt ratios (**P<0.01 PBS^CD^ vs PBS^RBD^, ***P<0.001 PBS^CD^ vs PBS^PD^, ****P<0.0001 *C*. *parvum*^CD^ or *C*. *parvum*^RBD^ vs *C*. *parvum*^PD^, ^*P*<0.05 PBS^CD^ vs. *C*. *parvum*^CD^, ^##^*P*<0.01 PBS^PD^ vs. *C*. *parvum*^PD^ (n = 3-7/group). (C) Growth through three days post-challenge with either *C*. *parvum* or Δ*C*. *parvum*. (N = 5-10/group, **P<0.01, ***P<0.001 (*C*. *parvum* vs. either PBS or Δ*C*. *Parvum*) and fecal parasite shedding (n = 5-10/group, *P<0.05, **P<0.01). (D) Immunofluorescence staining of epithelial cell tight-junction proteins (ZO-1, occludin, claudin-2) in ileum of CD and PD-fed infected mice and uninfected controls (n = 4/group). (E) Dose dependent persistent growth faltering (n = 10/group, *P<0.05, **P<0.01, ***P<0.001, ****P<0.0001 *C*. *parvum* 10^7^ vs *C*. *parvum* 10^6^; and ^####^*P*<0.0001 *C*. *parvum* vs PBS) and (F) fecal parasite shedding (**P<0.01, ****P<0.0001) through 21 days post-challenge. Data is representative of 2 replicate experiments.

The severity of *Cryptosporidium* infection in malnourished children directly correlates with quantitative fecal parasite burden [28], altered gut function [9], and growth impairments that persist beyond the period of active parasite shedding [11]. To optimize a malnutrition model that best recapitulated these features, we simultaneously performed *Cryptosporidium* challenge in weaned mice maintained on three different isocaloric diets: a full nutrient control diet (CD); a 7% protein, 5% fat, reduced vitamin diet (Regional Basic Diet-RBD) [29,30]; or a 2% protein, 15% fat, vitamin sufficient diet (isolated protein deficient-PD) [23, 24, 31] (Fig 1A). We found that 5 x 10^7^ *C*. *parvum* oocysts administered after 5 days of dietary acclimation led to weight loss and greater parasite shedding in PD-fed mice (Fig 1B). Severe zinc deficiency, which increases susceptibility to other enteric infections [32], did not further enhance the *C*. *parvum* infection phenotype (S1 Fig). Intestinal damage as measured by villus:crypt ratios was most significantly altered in *Cryptosporidium*-infected PD-fed mice (Fig 1B). Live oocysts were necessary to cause weight loss, confirming that the resultant pathology was due to active *C*. *parvum* infection and not a maladaptive response to parasite products in the malnourished host (Fig 1C).

Both the PD diet and *C*. *parvum* disrupted epithelial tight-junction expression. Specific tight-junction protein expression was measured using immunofluorescence four days after *C*. *parvum* challenge (Fig 1D). Neither expression nor localization of the tight junction scaffolding protein ZO-1 was altered under any of the conditions studied, providing a useful internal control. In contrast, occludin expression was reduced by PD alone. *C*. *parvum* infection also affected occludin, primarily by inducing redistribution to the intracellular vesicular pool. *C*. *parvum* infection with PD resulted in both reduced expression and enhanced internalization of occludin. As reported previously [33], little claudin-2 expression was present under basal conditions. This was not affected by PD alone. *C*. *parvum* infection upregulated claudin-2 expression, though only part of the claudin-2 expressed localized to the tight junction. In contrast, *C*. *parvum* infection with PD resulted in markedly increased claudin-2 expression, nearly all of which was concentrated at the tight junction. Finally, similar to the long-term growth impairments that are greater in symptomatic cryptosporidiosis than asymptomatic infections [10], challenge with 10^7^ *C*. *parvum* compared with 10^6^ *C*. *parvum*, led to more sustained concentration-dependent growth impairments through 21 days post-challenge (Fig 1E), despite a similar duration of shedding (Fig 1F).

### Protein malnutrition alters basal immune responses to *Cryptosporidium* antigens, while enhancing secondary responses following natural infection

**Fig 2:**
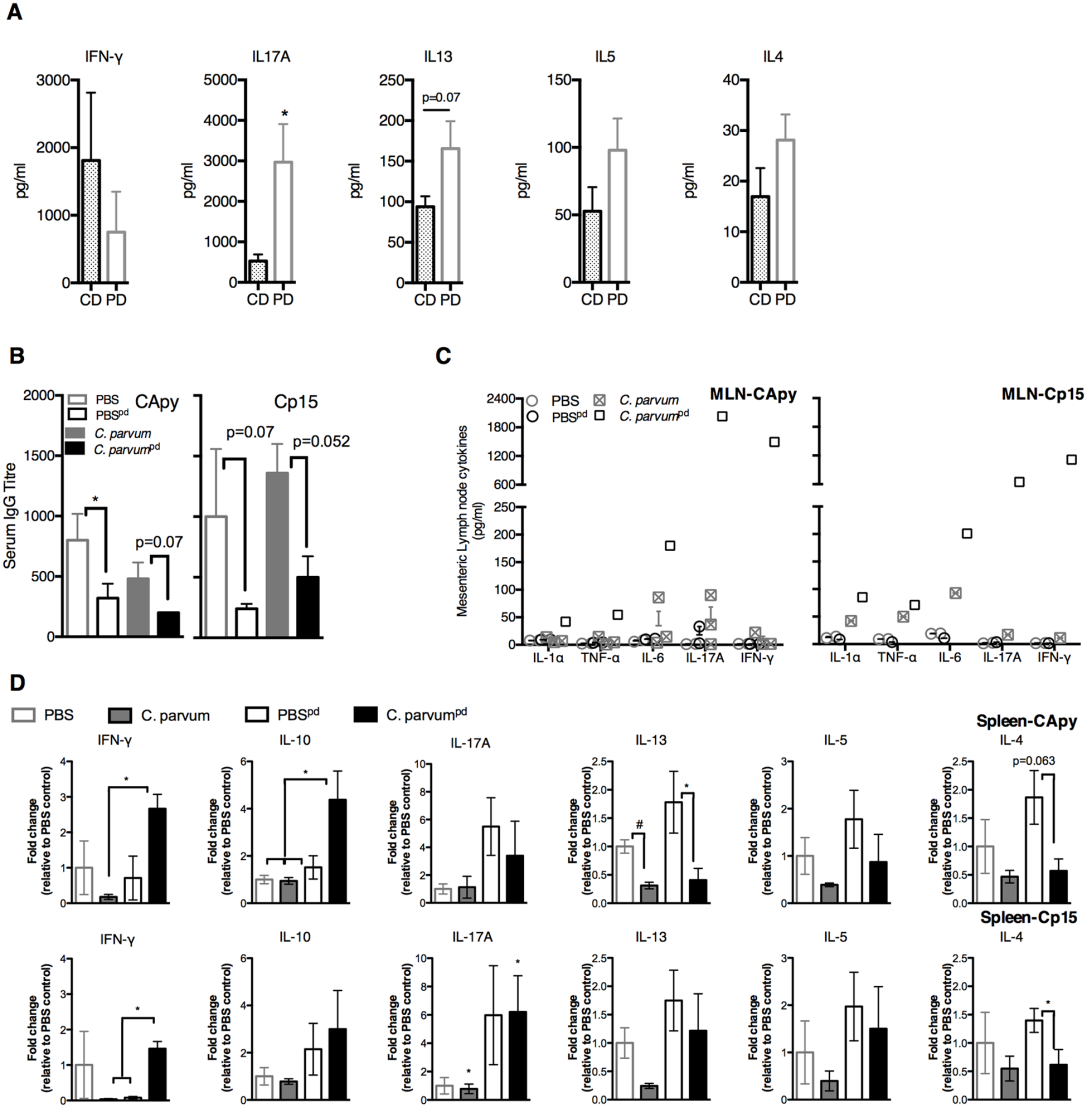
Protein malnutrition alters basal immune responses to primary *C*. *parvum* exposure, but secondary responses are intact. Immunologic responses to two different recombinant *Cryptosporidium* sporozoite antigens (CApy and Cp15) were performed at 13–15 days post *C*. *parvum* challenge in mice fed either control-diet (*C*. *parvum*^CD^) or protein-deficient diet (*C*. *parvum*^pd^) and results were compared with naïve age and diet-matched uninfected controls (PBS^CD^ and PBS^pd^). Mice began respective diets 12 days prior to *C*. *parvum* challenge and remained on the same diets post-challenge. (A) Cytokine secretion in splenocytes of naïve (uninfected) CD or PD-fed mic after stimulation with *Cryptosporidium* antigens. (B) Serum antibody production as anti-CApy or anti-Cp15 IgG titer (*P<0.05). (C) Cytokines secreted after CApy or Cp15 antigen stimulation in (C) mesenteric lymph nodes. (D) Cytokine secretion in splenocytes expressed as fold change relative to CD-fed uninfected controls. (*P<0.05 as indicated). Data is representative of pooled individual responses from two separate tissue harvests (n = 4-5/group).

We hypothesized that increased severity of cryptosporidiosis during PD was due to impaired Th1-type immunity. To compare basal immune responses to *Cryptosporidium* antigens during malnutrition, we stimulated splenocytes from uninfected CD or PD-fed mice (S1 Fig) with two different immunogenic recombinant *Cryptosporidium* sporozoite antigens (Cp15 and CApy) [34]. Primary responses to either *C*. *parvum* antigen were fundamentally different between naïve CD and PD-fed mice. Rather than IFN-γ, IL17A predominated in PD-fed mice along with a relatively IFN-γ and with tendency toward Th2-type cytokines (Fig 2A). Serum IgG titres were also constitutively lower in PD-fed mice (Fig 2B).

Secondary immune responses to either *Cryptosporidium* antigen at 13–15 days after *C*. *parvum* challenge were also diet-dependent, but strikingly opposite of the primary response in PD-fed mice. Nourished infected mice cleared parasites with little evidence of a serological response to either antigen. Serum IgG geometric mean titre (GMT) was attenuated both at baseline and after infection in all PD-fed mice (Fig 2C). Only the more heavily infected PD-fed *C*. *parvum* challenged mice, however, demonstrated robust secondary cytokine responses in mesenteric lymph nodes (MLNs), including IFN-γ (Fig 2C). Splenocytes of infected CD-fed mice mirrored the minimal cytokine responses in MLNs (Fig 2D). *C*. *parvum* challenged PD-fed animals unexpectedly demonstrated a 14- (CApy) and 41-fold (Cp15) increase in post-stimulation IFN-γ secretion, whereas the increases in IL-13, IL-5, and IL-4 seen in primary responses were reversed (Fig 2D).

### 10^6^ *C*. *parvum* protects against subsequent severe cryptosporidiosis and restores effector Th1-type immunity despite ongoing protein malnutrition

**Fig 3:**
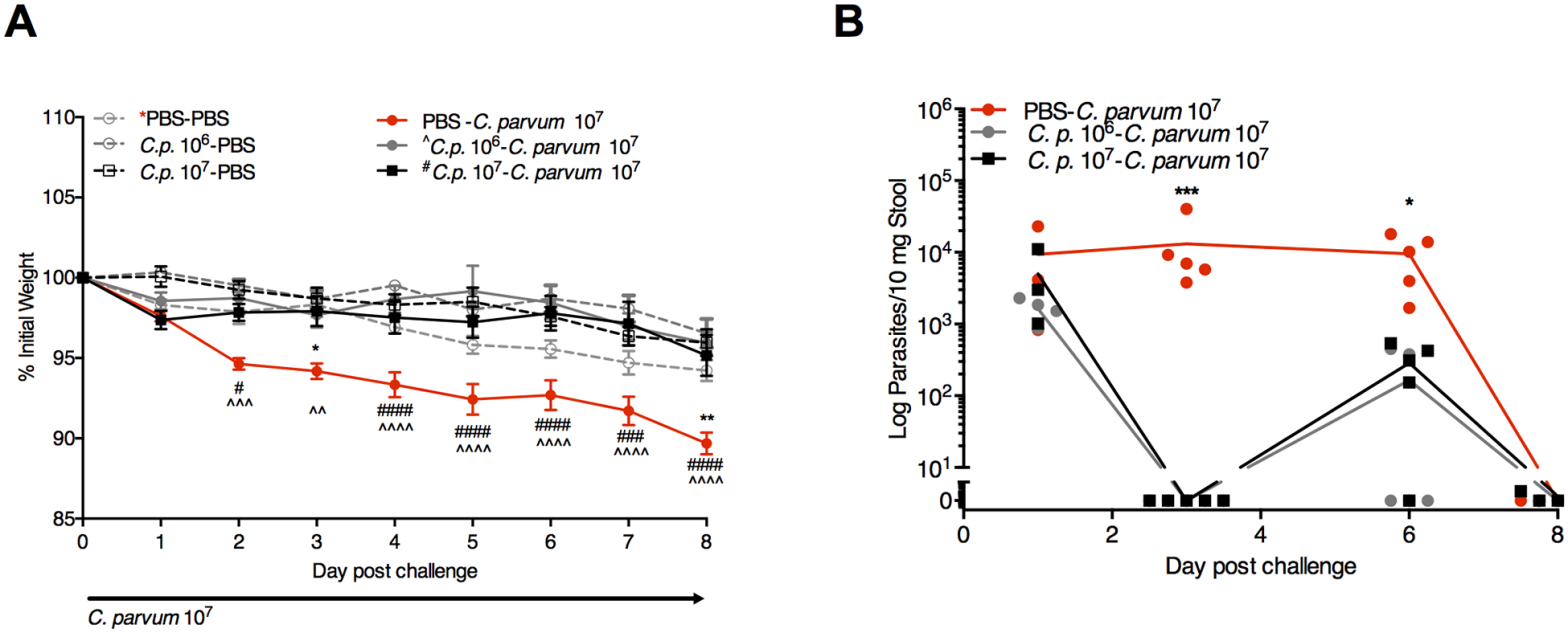
*C*. *parvum* priming protects against re-challenge despite continuous protein malnutrition. Growth (A) and parasite fecal shedding (B) following challenge with 10^7^ *C*. *parvum* oocysts in either naïve (PBS-*C*. *parvum* 10^7^) PD-fed mice, or mice previously exposed to either 10^6^ (Cp 10^6^) or 10^7^ (Cp 10^7^) *C*. *parvum* oocysts 21 days prior as indicated (n = 5/group). (A) *P<0.05, **P<0.01 for PBS-PBS vs PBS-*C*. *parvum* 10^7^, ^#^*P*<0.05, ^###^ *P*<0.001 ^####^ *P*<0.0001 for Cp 10^6-^*C*. *parvum* 10^7^ vs. PBS-*C*. *parvum* 10^7^, *^P*<0.05, and ^^*P*<0.01, ^^^^*P*<0.0001 for Cp 10^7-^*C*. *parvum* 10^7^ vs. PBS-*C*. *parvum* 10^7^. (B) *P<0.05, **P<0.01 for Cp 10^6-^*C*. *parvum* 10^7^ or Cp 10^7^-*C*. *parvum* 10^7^ vs. PBS-*C*. *parvum* 10^7^.

Since we previously observed decreased IFN-γ in ileal tissues of PD-fed mice during peak infection [23], we hypothesized that despite robust systemic IFN-γ recall following *C*. *parvum* challenge, PD would interfere with effective mucosal immune responses to serial *C*. *parvum* exposures. PD-fed mice were challenged with either a priming dose of 10^6^ *C*. *parvum* oocysts or the standard challenge dose of 10^7^ oocysts similar to re-challenge models in gnotobiotic piglets [35]. We confirmed clearance of parasite shedding within 11 days and through day 22 post-challenge in both groups prior to re-challenge with *C*. *parvum* 10^7^ oocysts on day 22 (Fig 1F). Despite ongoing PD, prior *C*. *parvum* exposure, unexpectedly, completely protected against weight loss (Fig 3A) and diminished parasite burden (Fig 3B).

#### Increases in mucosal CD8^+^CD103^+^ T-cells dominate the protective immune response to *C*. *parvum* re-challenge

**Fig 4:**
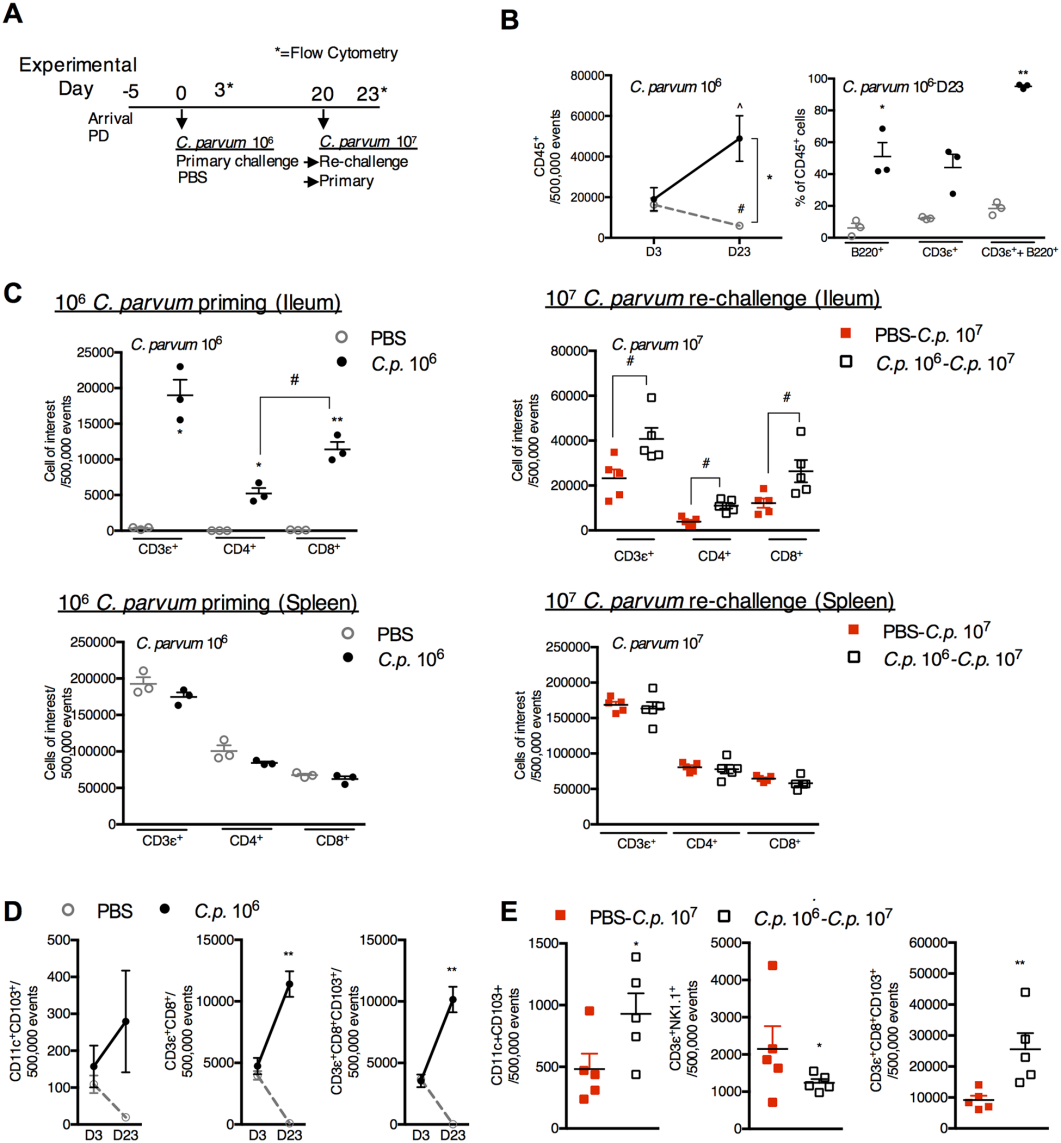
*C*. *parvum* priming promotes a progressive increase in CD3^+^CD8^+^CD103^+^ cells that further expand upon re-challenge. (A) Experimental timeline of 3 week-old C57Bl/6 mice initiated on the maintained on PD diet beginning 5 days prior to priming with *C*. *parvum* 10^6^ (●) or PBS control (grey◦). Day 0 is designated as the day of *C*. *parvum* priming. Mice were challenged with *C*. *parvum* 10^7^ 20 days later indicated as PBS-*C*.*p*. 10^7^ (red ◻) or *C*. *p*. 10^6^-*C*.*p*. 10^7^ (◻). Flow cytometry was obtained on day 3 (D3) and day 23 (D23) after *C*. *parvum* priming. (n = 3/group D3; n = 4-5/group D23). Day 23 also corresponds to 3 days after challenge with *C*.*p*. 10^7^. (B) Intestinal leukocytes in the ileum decrease between D3 and D23 in uninfected mice, but both B-cell (B220^+^) and T-cells (CD3ε^+^) increase in *C*. *parvum* primed mice. ^*P*<0.05 for *Cp* 10^6^ D3 vs *Cp* 10^6^ D23, #*P*<0.05 for PBS D3 vs PBS D23, *P<0.05 for PBS vs. *Cp* 10^6^. C) Expansion of T-cells in *C*. *parvum*-primed mice through D23 after *C*. *parvum* priming mice is specific to the ileum and accompanied by a predominance of CD8^+^ T-cells (left) that further increase 3 days after *C*. *parvum* re-challenge (right). *P<0.05 for PBS vs. *Cp* 10^6^, ^#^*P*<0.05 between groups as indicated. (D) CD11c^+^CD103^+^, CD3ε^+^CD8^+^ and CD3ε^+^CD8^+^CD103^+^ cells expand in the ileum through D23 after *C*. *parvum* priming. **P<0.01 for PBS vs. *Cp* 10^6^. (E) *C*. *parvum* priming leads to expansion of CD8^+^CD103^+^ cells and CD11c^+^CD103^+^ but not NK1.1+ cells compared with initial *C*. *parvum* 10^7^ challenge. *P<0.05, **P<0.01.

To determine whether *C*. *parvum* priming had restored Th1-type immunity despite ongoing PD, flow cytometry was performed on ileal tissues of PD-fed mice three days after 10^6^ *C*. *parvum* priming and again 23 days later (Fig 4A). At peak parasite shedding (day 3), total numbers of CD45^+^ cells (Fig 4B) and proportions of B-cells (B220^+^), T-cells (CD3ε^+^) and dendritic cells (CD11c^+^) were similar between both primed and naïve groups (S2 Fig). Although the CD45^+^ population progressively declined with ongoing PD, *C*. *parvum* priming dramatically expanded all CD45^+^ cells including both B-and T-cell populations (Fig 4B). CD8^+^ T-cells became predominant in the mucosal compartment at 23 days post-priming and corresponded with a modest but non-significant reciprocal decrease in splenic T-cells (Fig 4C, left). CD8^+^ T-cells also further increased in the ileum upon re-challenge, and a lack of reciprocal reductions in splenic T-cells suggested local expansion of this cell type (Fig 4C, right).

Further interrogation revealed that several cell-types associated with IFN-γ production (CD11c^+^ CD103^+^ dendritic cells, NKT (CD3ε^+^NK1.1^+^) cells, and CD8^+^ T cells) were depleted in the mucosa during prolonged PD, whereas, these cells progressively increased in *C*. *parvum* primed mice (Fig 4D). The majority of the CD3ε^+^CD8^+^ cells co-expressed the αE:β7 integrin, CD103^+^, confirming a mucosal imprinted phenotype (Fig 4D). Compared with 10^7^ *C*. *parvum* challenge in naïve mice, CD8^+^CD103^+^ cells in previously primed re-challenged mice increased more than 3-fold (Fig 4E).

#### 10^6^ *C*. *parvum* priming restores mucosal Th-1 type immune mediators during ongoing protein malnutrition

**Fig 5:**
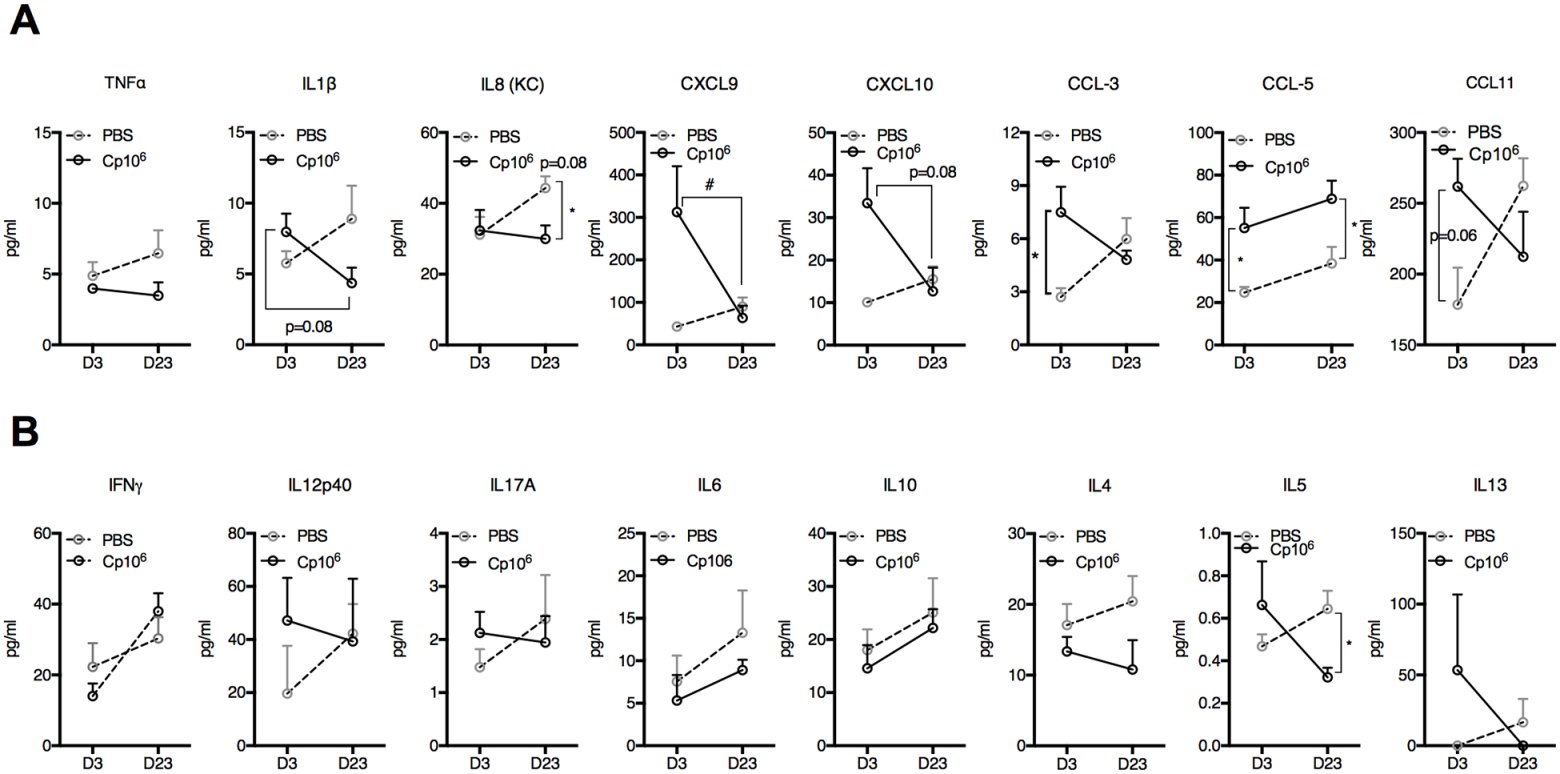
*C*. *parvum* priming leads to sustained changes in ileal tissue chemokine and cytokine profiles during protein malnutrition. Mice were conditioned on PD for 5 days prior to infection with 10^6^ *C*. *parvum* (Cp10^6^). Luminex was performed for measurement of chemokines and cytokines in ileal tissues at. day 3 (D3) and day 23 (D23) post challenge compared to uninfected controls (PBS) (n = 3-4/group). (A) Primary *C*. *parvum* infection led to increases in CXCL9, CXCL10, CCL-3, CCL-5, and CCL11 on D3. On D23, TNFα, IL1β, and IL-8 were diminished in infected mice relative to uninfected controls, however, CCL-5 continued to be elevated and other chemokines had returned to baseline. (B) Only IL12p40 and IL-13 were modestly elevated three days after primary *C*. *parvum* challenge. There was a relative decrease in all Th2-type cytokines through 23 days post-*C*. *parvum* compared with uninfected controls. (n = 3-4/group). *P<0.05 for PBS vs Cp10^6^ as indicated; #*P*<0.05 for Cp10^6^ D3 vs Cp10^6^ D23 as indicated.

Ileal chemokine and cytokine secretion at peak infection (day 3) and 23 days after exposure to *C*. *parvum* priming confirmed a restructured mucosal immune environment (Fig 5A). Although cell populations were identical between primed and naïve mice at day 3, CXCL9 and CXCL10 increased in *C*. *parvum* primed mice consistent with epithelial cell monolayer responses to *C*. *parvum* exposure [36,37] (Fig 5A). CCL-3, CCL-5 (aka RANTES-Regulated on Activation, Normal T-cell Expressed and Secreted), and CCL-11 were also elevated at day 3 in primed mice (Fig 5A). Only CCL5, however, remained significantly elevated through 23 days post-priming (Fig 5A). Similar to systemic primary splenocyte responses, mucosal IL-13, but not IFNγ, was elevated 3 days after priming (Fig 5B) [18]. Through 23 days post-priming, however, *C*. *parvum* exposure had remodeled mucosal inflammatory mediators. Otherwise progressive increases in TNFα, IL1β, IL-8 (CXCL1), CCL11 and Th2-type cytokines (IL-4, IL-5, and IL-13) (*P*<0.05 for IL-8 and IL-5) seen during prolonged PD in naïve controls were reversed in *C*. *parvum* primed mice (Fig 5A and 5B).

**Fig 6:**
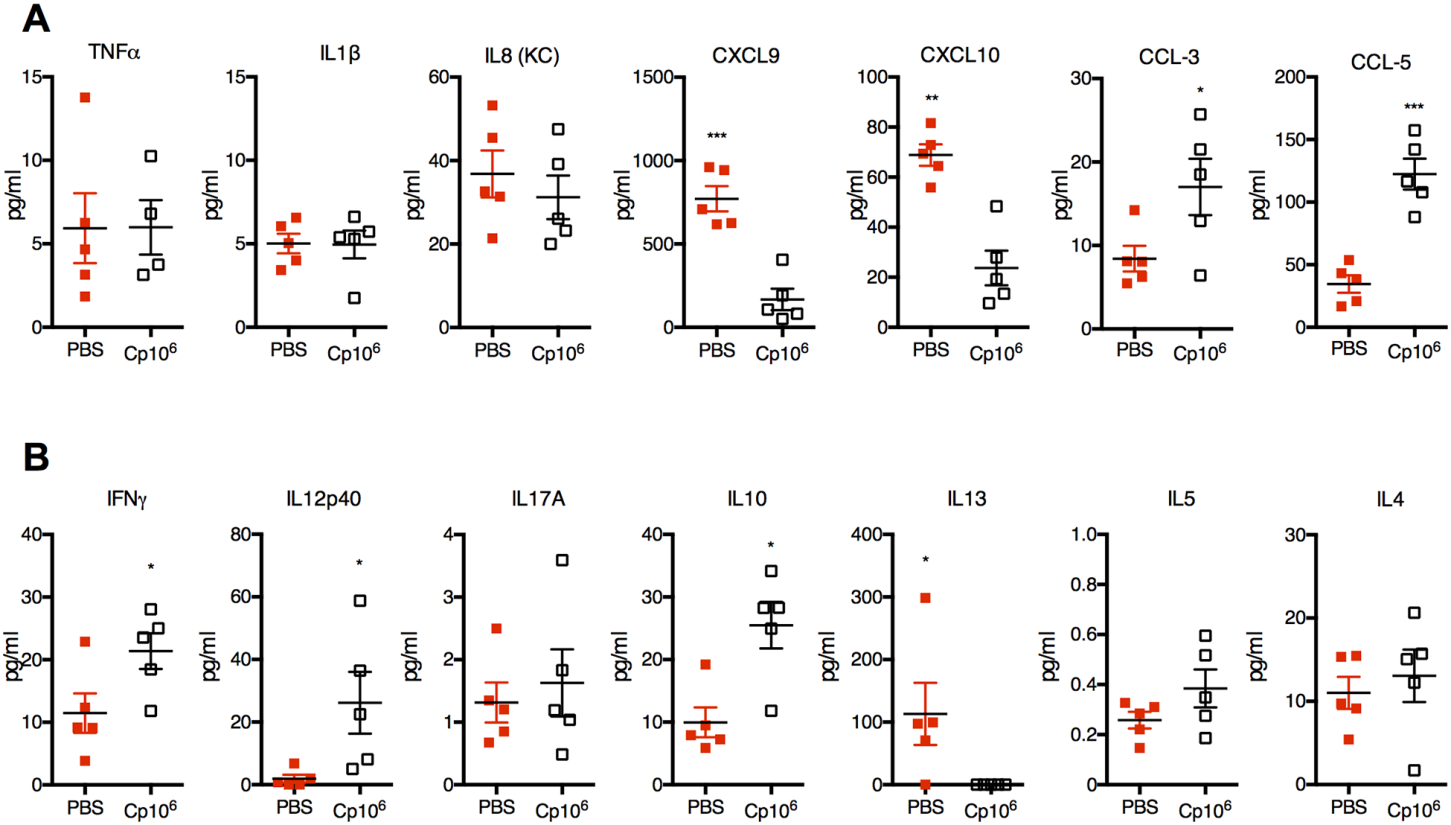
*C*. *parvum* priming enhances Th1-type cytokine responses to re-challenge in protein malnourished mice. (A) Ileal inflammatory mediators and chemokines and (B) cytokines measured three days after 10^7^ *C*. *parvum* challenge in previously uninfected (PBS) compared with mice primed with 10^6^ *C*. *parvum* (Cp10^*6*^) 20 days prior to re-challenge. *P<0.05, **P<0.01, ***P<0.001.

Compared with primary 10^7^ *C*. *parvum* challenge during prolonged PD (25 days), *C*. *parvum* primed PD-fed mice (Fig 4A) demonstrated robust elevations in CCL-3 and CCL-5 upon re-challenge (Fig 6A). IFN-γ, IL12p40, and IL-10 were all greater in secondary compared with primary challenge (Fig 6B). IL-13 was increased 100-fold during primary infection (Fig 6B), but was undetectable in secondary infection (*P*<0.05). Thus, coincident with the robust expansion of Th1-type effector cells (Fig 4D and 4E), *C*. *parvum* priming restored mucosal Th1-type cytokine responses despite ongoing PD.

### Vaccination strategies with *Cryptosporidium* antigens expressed in *S*. Typhi do not overcome protein malnutrition-induced susceptibility to *Cryptosporidium*, however *S*. Typhi alone partially reduces disease severity

#### Protein malnutrition interferes with vaccine-boosted immune responses to *Cryptosporidium* challenge

**Fig 7:**
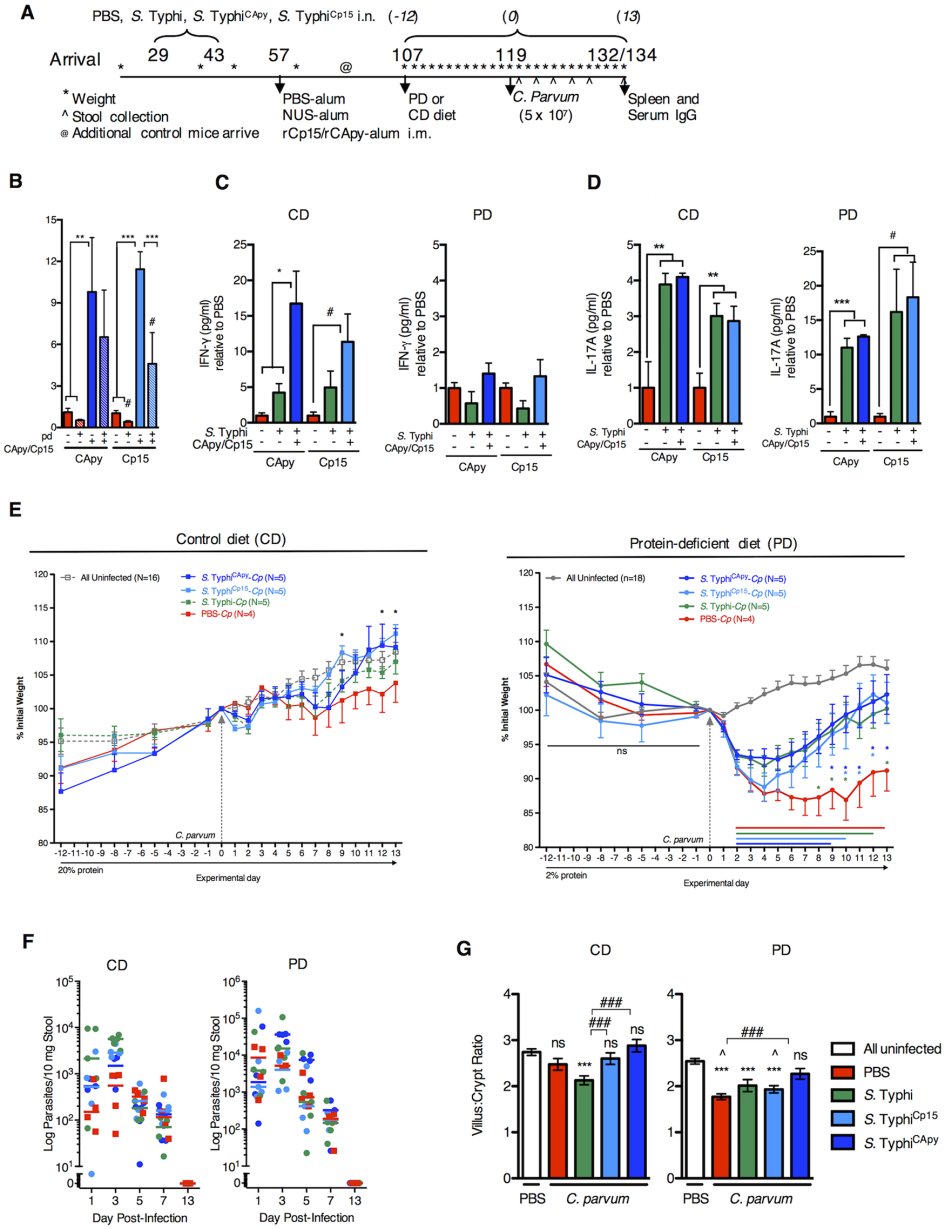
Protein malnutrition interferes with vaccine-boosted immunity, but the *S*. Typhi vector improves recovery after *C*. *parvum* challenge. (A) Timeline for immunization, growth monitoring, infection, and analysis of immune responses. 21 day-old mice acclimated for 4 days prior to weight-matched randomization (n = 8-19/group). Intranasal immunization with *S*. *enterica* Typhi 908*htr* vector expressing either of two recombinant sporozoite antigens, ClyA-Cp15 (*S*. Typhi^Cp15^ (aqua)) or ClyA-CApy (*S*. Typhi^CApy^ (blue)) was administered at two-week intervals. The *S*. Typhi vector alone (*S*. Typhi (green)) and a PBS-only (red) treatment served as a double-sham control. Intramuscular injection with rCp15 (*S*. Typhi^Cp15^), rCApy (*S*. Typhi^CApy^), the inert NUS peptide (for *S*. Typhi), or PBS-’sham’ (for PBS only group) combined with 1:1 alum adjuvant occurred two weeks after the second intranasal immunization. Serial weights (*) were obtained throughout the vaccination protocol (S1 Fig). On day 107 (9 weeks after *S*. Typhi exposure), mice were transitioned to either PD or CD diets (n = 4-10/group) and continued on respective diets throughout the remainder of the experiment. Mice were challenged with 5x10^7^ *C*. *parvum* (*Cp*) on day 119 and followed for 13–15 days post-challenge. (B) Serum geometric mean IgG titers (GMT) in *C*. *parvum* challenged groups. (C) IFN-γ and (D) IL-17A cytokine secretion recall responses to homologous vaccinogen as indicated. For (B-D), *P<0.05, **P<0.01, ***P<0.001, One-way ANOVA, Tukey post-test analysis, ^#^ *P*<0.05 by Student’s *t*-test. (E) Growth as percentage of weight change on the day of *C*. *parvum* infection beginning on the day of transition to either CD (left) or PD (right) diets. (left: *P<0.05, *S*. Typhi^Cp15^ vs PBS-*Cp*; right: colored bars indicate *P*<0.05 for PBS-*Cp* (red), *S*. Typhi-*Cp* (green), *S*. Typhi^Cp15^ (aqua), and *S*. Typhi^CApy^ (blue) vs. uninfected controls, *P<0.05 for individual vaccine groups [*S*. Typhi-*Cp* (green), *S*. Typhi^Cp15^ (aqua), and *S*. Typhi^CApy^ (blue)] vs. PBS-*Cp*. (F) Parasite shedding for infected groups: PBS (red), *S*. Typhi (green, *S*. Typhi^Cp15^ (aqua), and *S*. Typhi^CApy^ (blue). (G) Ileal villus:crypt for CD-fed (left) and PD-fed (right) mice in each *C*. *parvum* infected group or combined uninfected controls as indicated. Left: ***P<0.001 PBS-*Cp* vs uninfected controls, ^###^*P*<0.001 *S*. Typhi^Cp15^ or *S*. Typhi^CApy^ vs *S*. Typhi; Right: ***P<0.001 for PBS-*Cp*, *S*. Typhi, or *S*. Typhi^Cp15^ vs uninfected controls, ^###^*P*<0.001 *S*. Typhi^CApy^ vs PBS-*Cp*. ^*P*<0.05 for PD-PBS and PD-*S*. Typhi^Cp15^ vs DD-PBS and DD-*S*. Typhi^Cp15^. ns = not significant vs. uninfected controls.

We next determined whether the PM-associated immune deficiency could be overcome with other mucosal vaccine strategies. We previously established that humoral and cell-mediated recall responses to vaccine were preserved during PD, but vaccination was ineffective against *C*. *parvum* challenge [26]. We hypothesized, but had not previously tested, that PD therefore affected effector phases of vaccine responses at the time of *C*. *parvum* challenge, rather than development of memory during vaccination. To test this, we independently expressed either of two sporozoite antigens, the previously tested Cp15 and a novel calcium apyrase (CApy) vaccinogen, in the attenuated *S*. Typhi *908htr* vector system (*S*. Typhi^Cp15^ or *S*. Typhi^CApy^) as previously described [25,36]. Vaccine was delivered according to our optimized intransal prime-prime intramuscular boost strategy (Fig 7A). Immune responses and challenge studies were compared with the identical *S*. Typhi vector devoid of *Cryptosporidium* antigens as well as a ‘double-sham’ group of animals exposed only to PBS.

Mice were randomly allocated into four weight-matched groups (S3 Fig), and received the first intranasal treatment beginning on day of life 29. To distinguish vaccine-boosted immunity subsequent to *C*. *parvum* challenge from developing responses to recent vaccine exposure, both PD and *C*. *parvum* challenge were delayed according to the experimental protocol (Fig 7A). Serum IgG geometric mean titre (GMT) to homologous recombinant vaccinogens (*S*. Typhi^Cp15^ or *S*. Typhi^CApy^) in unchallenged mice remained detectable through 11 weeks post-vaccination [mean 0.48 (CApy) and 4.2 (Cp15)-fold change regardless of diet] (S3 Fig). Cytokine release in stimulated pooled MLNs of uninfected mice similarly confirmed cell-mediated responses to both vaccinogens (S3 Fig). Just as mice fed a PD-diet *during* the vaccination phase [26], transition to PD *after* vaccination also led to diminished IFNγ release in MLNs of *S*. Typhi^Cp15^ group. This post-vaccine PD effect was antigen-specific, however, and did not interfere with IFNγ release in the *S*. Typhi^CApy^ group (S3 Fig).

Despite waning of serum IgG GMT to either antigen at 11 weeks post-vaccination relative to our observations at earlier timepoints [26, 34], *C*. *parvum* challenge unmasked strongly boosted immunity in vaccinated mice (Fig 7B). PD, however, blunted the vaccine-boosted serum IgG effect (Fig 3B). Similarly, post-challenge splenocyte IFN-γ responses in vaccinated CD-fed were markedly enhanced compared with natural infection, but not in the PD-fed vaccinated mice (Fig 7C). In contrast, IL-17A responses dominated in the vaccinated PD-fed mice (Fig 7D). Irrespective of diet, IL17A responses were driven by exposure to the *S*. Typhi vector rather than either *Cryptosporidium* antigen. Real-time PCR for *S*. Typhi at 24 hours after each intranasal exposure and again 24 hours prior to the next prime/boost challenge confirmed exposure to the vector was transient (S4 Fig).

#### Remote *S*. Typhi vector exposure non-specifically facilitates post-infection recovery

Although disease was mild in the CD-fed groups, *S*. Typhi^Cp15^ or *S*. Typhi^CApy^ vaccinated mice demonstrated a relative growth improvement, compared with challenge in ‘double-sham’(PBS)-exposed animals. This 5.8–7.7% relative growth advantage reached significance on days 8, 12, and 13 post-infection in the *S*. Typhi^Cp15^ group (Fig 4E, left), but appeared to be partially driven by any *S*. Typhi exposure. Similarly, in PD-fed mice, any *S*. Typhi exposure, independent of *Cryptosporidium* antigen expression, partially mitigated weight loss (nadir 8.7% weight loss on day 3 for aggregated *S*. Typhi groups vs 13.1% on day 10 in the sham-exposed group, S4 Fig), and promoted growth recovery (Fig 3E, right).

Within each diet cohort, *C*. *parvum* shedding was similar across all experimental groups (Fig 7F). Intestinal damage as measured by decreased villus:crypt (V:C) ratios was worse in all PD-fed *C*. *parvum* challenged mice compared with diet-matched uninfected controls. Whereas in CD-fed mice, either *S*. Typhi^CApy^ or *S*. Typhi^Cp15^ reduced intestinal damage compared with *S*. Typhi alone (Fig 7G), in PD-fed mice only *S*. Typhi^CApy^ reduced intestinal damage. Thus, vaccination with either *S*. Typhi^Cp15^ or *S*. Typhi^CApy^ in the CD-fed mice was effective at boosting post-infection immune responses and diminishing disease severity without an apparent reduction in *C*. *parvum* shedding. PD attenuated the vaccine-boosted IgG and IFN-γ responses to infection, but amplified the over-exuberant IL17A response driven by any *S*. Typhi exposure. In PD-fed mice any aggregated remote *S*. Typhi exposure facilitated post-infection recovery with a modest decrease in *C*. *parvum* shedding (S4 Fig).

### Priming with viable *C*. *parvum* oocysts is more effective than remote intranasal exposures to the non-specific immune activators CpG and *S*. Typhi

In subsequent experiments, even in the absence of *Cryptosporidium* antigen expression, intranasal *S*. Typhi vector exposures at 6 and 4 weeks prior to 10^7^ *C*. *parvum* challenge partially reduced disease severity (S4 Fig). The effect was likely not due to *S*. Typhi-enhanced IL17A responses. While *IL17* expression in the ileal mucosa remained elevated several weeks after *S*. Typhi exposure in some mice, IL17RA mice, which lack IL17 signaling, were relatively less susceptible to *C*. *parvum* challenge than wild-type controls (S4 Fig). Rather, the benefit of *S*. Typhi more resembled the partial benefit seen in PD-fed mice receiving an intraperitoneal TLR9 agonist CpG-ODN 1668 at the time of infection (S5 Fig). Intriguingly, remote intranasal CpG, though incompletely protective, was more effective than intraperitoneal CpG delivered at the time of infection. There was no apparent additional benefit to *S*. Typhi combined with CpG (S5 Fig).

**Fig 8:**
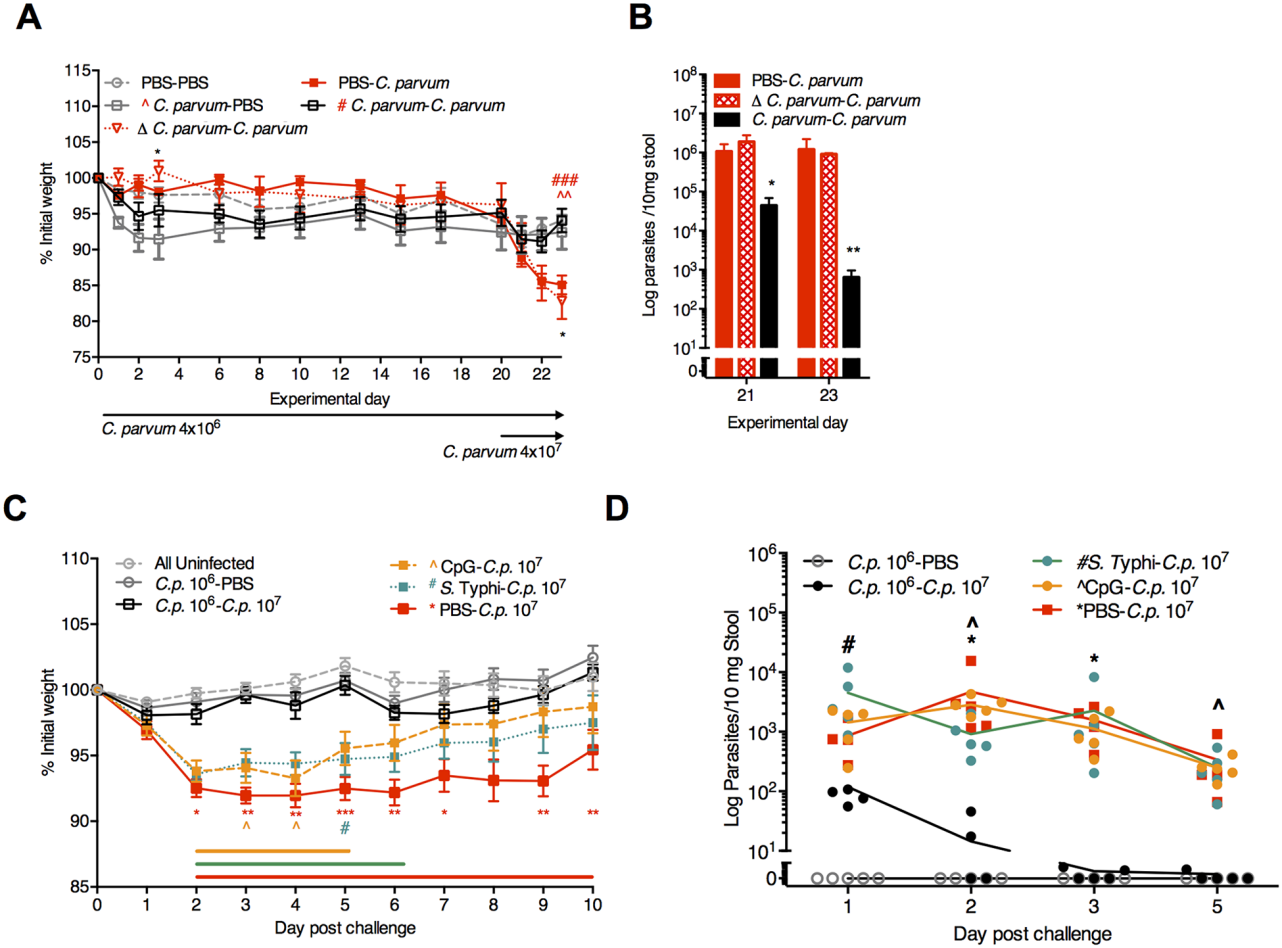
Viable *C*. *parvum* priming provides greater protection against re-challenge than either CpG-ODN or *S*. Typhi. (A, B) Comparison of protective immunity following priming with either viable and heat-inactivated (Δ) *C*. *parvum* 10^6^. (A) Growth of PD-fed mice through 23 days post-priming with either 4x10^6^ viable (*C*. *parvum*) or 4x10^6^ heat-inactivated (Δ*C*. *parvum*). Mice were challenged with viable 4*x*10^7^ *C*. *parvum* oocysts on day 20 post-priming. *P<0.05 for Δ*C*. *parvum-C*. *parvum* vs. *C*. *parvum*-*C*. *parvum* (d3 and d23); ^^*P*<0.01 for *C*. *parvum*-PBS vs PBS-*C*. *parvum* (d23); ^###^*P*<0.001 for *C*. *parvum*-*C*. *parvum* vs. PBS-*C*. *parvum* (d23). (B) RT-PCR of *Cryptosporidium* stool shedding on experimental days 21 and 23 (day 1 and day 3 after *C*. *parvum* 10^7^ challenge, respectively). *P<0.05 and *P<0.01 for *C*. *parvum*-*C*. *parvum* vs either PBS-*C*. *parvum* or Δ*C*. *parvum-C*. *parvum*. (C,D) 3-week-old C57Bl/6 mice were conditioned on PD for 7 days prior to orogastric inoculation with 10^6^ *C*. *parvum*, intranasal (i.n.) 10^9^ *S*. Typhi 908*htr*, i.n. CpG-ODN 1668 (100 mcg), or PBS (100 mcl) as indicated (n = 10/group). On day 21, mice were re-challenged with either PBS or *C*. *parvum* 10^7^. (C) Growth as percentage of initial weight, normalized to the day of 10^7^ *C*. *parvum* challenge (Day 0). The group labeled “All uninfected” includes animals that received either PBS during both inoculations, CpG followed by PBS, or *S*. Typhi followed by PBS (n = 5/group x 3 = 15) given all three groups grew similarly and were never exposed to *C*. *parvum* (S4 Fig). *P<0.05, **P<0.01, ***P<0.001 for PBS–*C*.*p*.10^7^ (red) vs *C*.*p*.10^6-^*C*.*p*.10^7^, ^*P*<0.05 for CpG-*C*.*p*.10^7^ (yellow) vs *C*.*p*.10^6-^*C*.*p*.10^7^, and ^#^*P*<0.05 for *S*. Typhi-*C*.*p*.10^7^ (green) vs *C*.*p*.10^6-^*C*.*p*.10^7^). Horizontal lines designate significant differences at *P*<0.05 between CpG-*C*.*p*.10^7^ (yellow), *S*. Typhi-*C*.*p*.10^7^ (green), and PBS-*C*.*p*.10^7^ (red) vs. All uninfected controls, respectively. (D) Parasite fecal shedding in serial fecal pellets collected on indicated experimental days post *C*. *parvum* 10^7^ challenge. *P<0.05 for PBS–*C*.*p*.10^7^ vs. *C*.*p*.10^6-^*C*.*p*.10^7^, ^*P*<0.05 for CpG-*C*.*p*.10^7^ vs *C*.*p*.10^6-^*C*.*p*.10^7^, and ^#^*P*<0.05 for *S*. Typhi-*C*.*p*.10^7^ vs *C*.*p*.10^6-^*C*.*p*.10^7^. Data is representative of two replicate experiments.

Since either CpG-ODN in PD-fed weanling mice [24] or oral CpG in neonates [38] restores mucosal IFN-γ, we hypothesized that remote intranasal CpG might be as efficacious as *C*. *parvum* priming. To test this hypothesis we first confirmed that only viable *C*. *parvum* priming, but not heat-inactivated *C*. *parvum*, established protective immunity (Fig 8A and 8B) and increased Th1-type cytokines (S6 Fig). While even a single mucosal exposure to intranasal CpG-ODN 1668 (CpG*-C*. *parvum*) and even *S*. Typhi (*S*. Typhi-*C*. *parvum*) 21 days prior to *C*. *parvum* challenge facilitated post-infection recovery, only the single 10^6^ *C*. *parvum* priming afforded complete protection against weight loss and rapid parasite clearance (Fig 8C and 8D; S5 Fig). Thus, while enhancing host defenses through transient exposures during PD has a lasting impact on cryptosporidiosis severity, only *C*. *parvum* priming, which activated secondary Th1-type effector responses, resulted in protective immunity.

## Discussion

**Fig 9:**
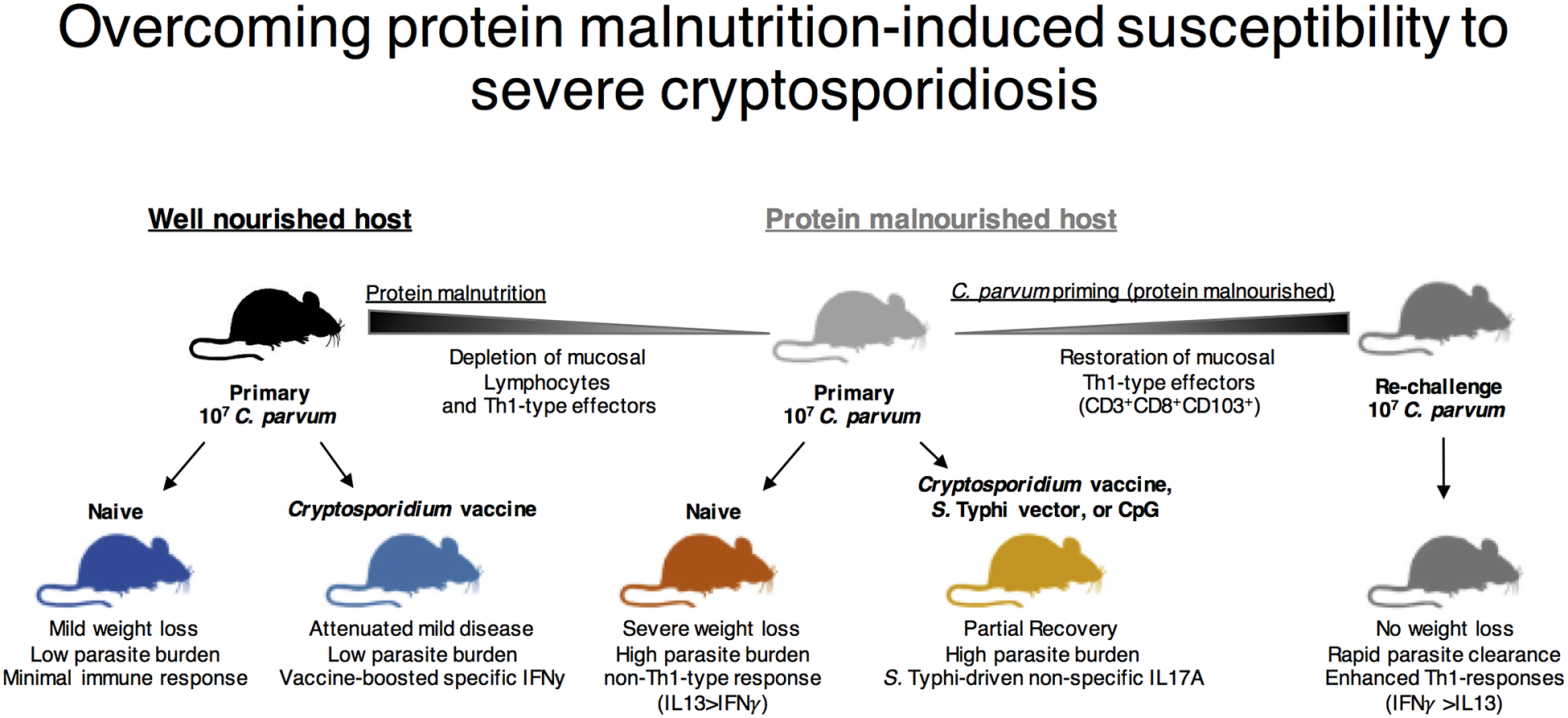
Schematic of non-specific (*S*. Typhi and CpG) and specific (*C*. *parvum* priming) mucosal exposures that modulate host immunity and protect against cryptosporidiosis during malnutrition. Strategies to enhance immune defenses against *Cryptosporidium* infection during malnutrition were investigated in a protein deficient murine model that replicates clinical features of childhood cryptosporidiosis. Whereas the well-nourished host (black) clears *Cryptosporidium* with little evidence of a secondary immune response (dark blue), mucosal vaccination with *Cryptosporidium* antigens expressed in an *S*. Typhi vector can elicit strongly boosted IFNγ-predominant immune responses to subsequent challenge (light blue). Vaccine attenuates the mild disease caused by *Cryptosporidium* in well-nourished hosts. In protein malnourished hosts (light grey) there is ongoing depletion of mucosal lymphocytes including Th1-type effectors. This results in enhanced disease after primary *C*. *parvum* challenge with a response characterized by decreased IFNγ but increased IL13 and tendency toward Th2-type cytokines (red). Unlike in nourished hosts, vaccine does not further enhance IFNγ to primary *C*. *parvum* challenge, but rather the *S*. Typhi vector alone drives increased IL17A and partially attenuates disease severity similar to the TLR9 agonist CpG (yellow). *C*. *parvum* priming, however, leverages a robust secondary Th1-type response to *C*. *parvum* during protein malnutrition, and even at low-doses in this model establishes a mucosal imprinted population of CD8^+^ T-cells along with protective immunity to subsequent re-challenge (dark grey).

The epidemiology of malnutrition and *Cryptosporidium* are intimately associated, but mechanisms whereby malnutrition increases severity of cryptosporidiosis are poorly understood. In order to improve understanding of how malnutrition-induced mucosal immune deficits influence *Cryptosporidium* infection and immune responses, we isolated which nutritional deficiencies have the greatest impact on mucosal host defenses against *Cryptosporidium*. We then determined which of several anti-cryptosporidial preventive strategies would most effectively overcome the resultant immunodeficiency. Protein malnutrition (PM) in mice selectively replicated clinical, histological, and immunological features of active *Cryptosporidium* infection in malnourished children. Using remote exposures to non-specific mucosal defense enhancers (*S*. Typhi vaccine vector and CpG), vaccination with *Cryptosporidium* antigens expressed in an *S*. Typhi vector, and viable *C*. *parvum* priming we demonstrated that while PM fundamentally alters Th1-type basal and vaccine-boosted immune responses at the time of infection, the resultant immunodeficiency is not insurmountable. Rather, robust and protective adaptive Th1-type effector responses can occur when priming with viable *C*. *parvum*. These findings, summarized in Fig 9, provide new insights into nutrient-dependent mucosal immune deficiencies relevant to *Cryptosporidium* outcomes and a viable model in mice for future comparisons of *Cryptosporidium* prevention strategies.

*Cryptosporidium* is a challenging parasite to study and there is a lack of established models that confidently mimic human disease. Thus, we first wanted to establish which model would best recapitulate known clinical features of active *Cryptosporidium* infection in malnourished children. While we and others have used various malnutrition protocols to intensify enteric infections [23, 24, 31, 29, 39] it was not known which nutrients were most essential for *Cryptosporidium* susceptibility. Only isolated protein malnutrition, but not multinutrient deficiency, sufficiently replicated dose-dependent disease severity evidenced by weight loss and persistent growth impairment, intestinal villus injury and epithelial tight junction disruption, and the elevated IL13 but paradoxically diminished IFNγ response seen in malnourished children. Amino acid dependent immune pathways therefore may be most relevant for *Cryptosporidium* outcomes. In contrast, isolated zinc deficiency less markedly enhanced cryptosporidiosis even though zinc can shorten duration of diarrhea and lessens severity and virulence of enteroaggregative *Escherichia coli* infection in our models [32]. Indeed, current standard WHO-recommended oral rehydration therapy and zinc treatment appears to be less effective for cryptosporidiosis than other diarrheal pathogens in children [6]. Rather, targeted amino acid therapies such as alanyl-glutamine [24, 40] or arginine [41] may more selectively diminish severity of *Cryptosporidium* infection.

Prolonged PM in our model, like total caloric restriction [42], leads to progressive depletion in multiple IFNγ-producing effector cell populations (CD8+ T-cells, CD11c^+^CD103^+^ dendritic cells, and NKT cells) that should otherwise be steadily increasing in maturing young mice [43]. Consequently, like in malnourished children with active cryptosporidiosis [18], either peak primary *C*. *parvum* challenge during PM in mice or stimulation of their naïve lymphocytes with *C*. *parvum* antigens elicits a basal IL13 response rather than IFNγ. Consistent with this observation, a systematic review concluded that malnutrition most significantly impairs effector T-cell responses and Th1-type cytokines but not total T- and CD4^+^ T-cell numbers. Malnutrition also associated with a tendency toward Th2-type cytokines [2]. We find that PM alone is biologically sufficient to recapitulate a similar immunological alteration. These data contribute to increasing recognition that immune effector function is differentially altered, rather than globally suppressed, by select nutrient deficiencies. Vitamin A deficiency, for example, disproportionately alters ratios but not absolute numbers of mucosal innate lymphoid subsets, increasing susceptibility to *Citrobacter rodentium* while paradoxically enhancing immunity against intestinal nematodes [44].

In the case of *Cryptosporidium* infection, a skewed response during PM may not only increase susceptibility to primary infection, but also contribute to the pathogenesis. Our studies confirm for the first time *in vivo* tight-junction disruption seen in *in vitro Cryptosporidium* models [45, 46]. Of the studied epithelial cell proteins, the most marked disruptions were seen in the cytokine-inducible tight-junction proteins occludin and claudin-2. Both internalization of occludin and IL-13 induced claudin-2 upregulation have also been reported in human and experimental inflammatory bowel disease models [33, 47]. Internalization of occludin occurs during acute TNF-α induced barrier loss and diarrhea [48]. *In vitro* and *in vivo* studies have shown that removal of occludin from the tight junction, either by endocytosis or reduced expression, facilitates paracellular flux of small and large molecules via the ‘leak pathway’. This complements the increased ‘pore pathway’ permeability to water and small cations following increases in claudin-2 expression [49]. In addition to potentially promoting diarrhea, the inflammatory response to *Cryptosporidium* during PM corresponds with persistent growth impairment, and may resembles the chronic T-cell activation seen in the environmental enteropathy associated with malnutrition [50]. Thus, not only heavier *Cryptosporidium* infection, but potentially aberrant host responses during malnutrition contribute to the greater severity of diarrhea and subsequent post-infection sequelae.

While PM-induced depletion of several cell types may be independently important for anti-cryptosporidial defenses, CD11c^+^CD103^+^ cells that co-express the CXCR3 receptor were recently shown to be more consequential for early *Cryptosporidium* clearance in primary infection in neonatal mice than loss of either NK cells or T-cells [43]. Although CD11c^+^CD103^+^ cells did increase following *C*. *parvum* priming in the current study, the influx of CD3^+^CD8^+^ T-cells was even more striking. Sensitized CD8^+^ T-cells have a direct role in clearing *Cryptosporidium* from human epithelial cells [51] as well as in experimental models of *C*. *muris* infection [52, 53] and have been shown to accumulate after infection in neonatal mice [54, 55]. Preceding the influx CD3^+^CD8^+^ T-cells in this model was an increase in Th1-type chemoattractants CXCL9, CXCL10, CCL3 and CCL5 at peak primary infection. Epithelial cells are a major source of these chemokines in response to exposure to *Cryptosporidium* [43, 56, 57], and appear to be functional despite PM. Interestingly, however, CCL5 but not CXCL9 or CXCL10, dominated re-challenge responses during PM. This is in contrast with the predominance of CXCL10 rather than CCL5 in patients with HIV/AIDS during *Cryptosporidium* infection, and is consistent with the likelihood that the influx of T-cells after *C*. *parvum* priming in this model contributed to elevated CCL5 [56]. Ours is the first study to identify that the majority of CD3^+^CD8^+^ also co-express the αE:β7 marker CD103^+^ indicative of intestinal honing [58] in response to *Cryptosporidium*. A subpopulation of these cells has recently been classified as tissue resident effector memory (Trm) cells that produce higher amounts of IFNγ than CD3^+^CD8^+^CD103^-^ cells, accumulate locally following intracellular infections at mucosal sites [59, 60] and thus represent an emerging target for mucosal vaccine development [61, 62]. Further elucidation of the role of Trms in *Cryptosporidium* infections may therefore further inform vaccine development [13].

Together with our previous findings [26], our collective vaccine studies show that PM during the priming phase of intranasal vaccination does not impair immunogenicity to *Cryptosporidium* antigens expressed in an *S*. Typhi vector, but PM at the time of infection impairs vaccine-boosted immunity and does not protect against *Cryptosporidium* challenge. Similar effects were seen during PM and a multistage antigen-based *Mycobacterium tuberculosis* vaccine-challenge model in mice [63]. Since our investigation began, it has been recognized that an oral route may more optimally target vaccine responses to the small intestine than the intranasal route used in our studies, including *Cryptosporidium* antigens expressed in an attenuated *S*. Typhimirium vector [61, 64]. Thus, oral rather than intranasal *Cryptosporidium* vaccine delivery might better mimic *C*. *parvum* priming. Intranasal vaccination with either *Cryptosporidium* antigen in fully nourished hosts leads to a robust enhancement in the immune response, similar to human volunteers that demonstrated serum IgG antibodies only after secondary, but not primary *C*. *parvum* infection [65]. As fully nourished mice appear to clear *C*. *parvum* infection via innate defenses, there was no apparent decrease in parasite clearance following vaccination. These findings raise important implications in the limitations of inferring correlates of immunity in healthy volunteers and desired effective responses in malnourished target populations. Similarly, Maier et al. demonstrated that malnourished mice infected with rotavirus demonstrated diminished seroconversion, but were unexpectedly protected against viral re-challenge [66].

We present a series of experiments designed to compare the effect of both specific (sporozoite antigen vaccine and homologous re-challenge) and non-specific (the attenuated *S*. Typhi vector and the TLR9 agonist CpG) antigen exposures. In this model, we demonstrate that while less effective than *C*. *parvum* priming, even remote non-specific exposures can diminish disease severity, even if not reducing parasite shedding. Others have reported partial efficacy of vector-based anti-*Cryptosporidium* vaccines in animal models [67], including apparent unexplained phenomena of the vector alone [68, 69]. In the present study *S*. Typhi enhanced IL-17A production, most strikingly during PM. IL-17A responses are also increased in naturally acquired *S*. Typhi [70] and Ty21a vaccination [71]. While the loss of IL-17A producing lymphocytes promotes disease severity in simian immunodeficiency virus and *Cryptosporidium* co-infection [72] a protective role of IL-17A against *Cryptosporidium* remains undefined. Alternatively, we speculate that *S*. Typhi provided broad activation of innate immunity and thus modulated mucosal responses to microbial products such lipopolysaccharide [26], or influenced claudin protein expression and barrier function [33, 47, 73, 74]. Oral immunization with *Salmonella* vaccine strains has also been shown to protect against non-*Salmonella* bacterial infections via sustained changes in TLR expression and non-specific macrophage activation [75]. Signaling TLR3 in combination with TLR5 [76, 77], TLR4 [78] and TLR9 [24, 38] independently influence *Cryptosporidium* infection outcomes. While prior studies have focused on TLR activation immediately prior to infection, we found that even remote selective TLR activation with CpG mimicked the *S*. Typhi effect and was more effective than CpG administration immediately prior to infection. Such observations are aligned with the concept of “trained innate immunity” with potential for enhanced responses to repeated exposures to non-specific microbial products [79]. These concepts may be highly relevant in malnourished children given potentially divergent microbiota and frequent exposures to enteropathogens associated with childhood malnutrition [80].

Important questions remain regarding primary and secondary immune responses to *Cryptosporidium* in malnourished children. First, although many features of our model overlap with childhood cryptosporidiosis during malnutrition, murine immunology is not a surrogate for children, and further investigations are needed to delineate whether malnourished children similarly demonstrate such contrasting immune responses to primary and secondary infection. Indeed, despite our finding robust protective immunity after even low-inoculum *C*. *parvum* exposure, recurrent and multiple *Cryptosporidium* infections are well documented in malnourished children [8,81]. The prevalence of asymptomatic infections is only beginning to be defined as detection of these exposures may require highly sensitive molecular diagnostics [28], and when applied outnumber diarrheal infections ~10:1 [12]. If asymptomatic exposure does induce a protective response, the duration of immunity in children may be short lived. While recurrence was unlikely within one month of infection in one cohort [82], by nine weeks post-infection only 54% of children in a different study demonstrated antibody to an immunodominant *Cryptosporidium* antigen gp15 through nine weeks [83]. Although humoral and cell-mediated responses to specific *Cryptosporidium* antigens are not universally concordant [20, 84], these findings suggest that immunity in children may rapidly wane. Also, our findings are restricted to homologous *C*. *parvum* re-challenge and may as such be strain-specific. We did not have access to *C*. *hominis* and important anthroponotic strains of *Cryptosporidium parvum* [5, 85] that when given to gnotobiotic piglets reveal incomplete heterologous protection [35]. This is important since sequential infections with different *Cryptosporidium spp*. subtype are documented [82,86]. Finally, the role of passive maternal immunity in infants [87] and the role of intestinal microbiota remain important considerations not addressed in the present study.

In conclusion, in a PM model that replicates several clinical and immunologic features of *Cryptosporidium* infection in malnourished children, our findings raise important future directions for understanding *Cryptosporidium* pathogenesis and immunity during malnutrition. First, the stark contrasts in immune responses in natural *C*. *parvum* infection and post vaccine-boosted immunity during PM compared with fully nourished animals reinforces the need for further investigation to distinguish primary from secondary responses to *Cryptosporidium sp*. in malnourished children. Second, elucidating the independent roles of direct consequences of *Cryptosporidium*-induced damage from a potentially deleterious host inflammatory response on tight-junction alterations may advance understanding of cryptosporidiosis pathogenesis and raise novel candidate therapeutics. Finally, since alterations in basal immune responses during malnutrition appear to have the greatest impact at the time of infection, a successful anti-*Cryptosporidium* vaccine for malnourished children may need to consider not only systemic correlates of immunogenicity, but a careful examination of which mucosal effector responses are most indicative of future protection. Thus, overcoming defective mucosal T-cell effector responses, such as enriching intestinal CD3^+^CD8^+^CD103^+^ cells population, may be a component of successful strategies to eliciting protective anti-cryptosporidial immunity during malnutrition.

## Methods

### Ethics statement

This study included the use of mice. This study was conducted in strict accordance with recommendations in the Guide for the Care and Use of Laboratory Animals of the National Institutes of Health. The protocol was approved by the the International Animal Care and Use Committee at the University of Virginia (Animal Care and Use Committee Protocol number: 3315). Tissue procurement was performed under anesthesia that was induced and maintained with ketamine hydrochloride and xylazine, and all efforts were made to minimize suffering.

### Animals and malnutrition

All animal experiments were performed at the University of Virginia (UVa). Weaned 21-day-old female C57Bl/6 wild-type mice were purchased from Charles River for all experiments. On arrival, mice were acclimated for at least 48 hours prior to handling. Mice were randomly distributed in weight-matched groups prior to any interventions. Experimental isocaloric diets consisted of either: a full nutrient (20% protein) control diet (CD) (TD.08678, Harlan; or Research Diets), a multinutrient deficient (7% protein, 5% fat, vitamin reduced diet) Regional Basic Diet (RBD, Research Diets), a 2% protein, 15% fat, vitamin sufficient, isolated protein deficient diet (PD) (TD.08679, Harlan), or isolated zinc deficient but otherwise full nutrient diet (ZD) (Research diets). Diets were either upon arrive of mice to the facility, or for *S*. Typhi vaccine experiments at protocolized timepoints prior to infection. All mice remained on their respective diets diets throughout the remainder of the experiment. Mice in experiments designed for testing boosted vaccine responses were originally on in-house chow (Harlan) prior to transitioning to respective diets between 5–12 days prior to *Cryptosporidium parvum* infection, a range that shows a consistent phenotype in this model [23, 24]. IL17RA^-/-^ mice on a C57Bl/6 background were obtained through a material transfer agreement with Amgen.

### *Cryptosporidium parvum* infection

Oocysts of *C*. *parvum* (Iowa isolate) were purchased from Waterborne, Inc. (New Orleans, LA) at a concentration of 1 x 10^9^/50 mL PBS. Oocysts were rinsed in PBS using centrifugation at 650 *g* prior to infection [24]. The final concentration of the challenge inoculum was determined using a hemacytometer and the pellet was re-suspended in PBS to yield a final concentration of 1–5 x 10^7^ oocysts in 100 μl or as a 1:10 dilution (1–5 x 10^6^ oocysts/100 μl) for low-dose inoculum experiments. For experiments using heat-inactivated *Cryptosporidium*, lot-matched oocysts were placed in a dry block shaker (Thermomixed, Eppendorf) at 90°C for 10 minutes with 300 rpm shaking per prior protocols [88].

### Histology and immunofluorescence staining

Ileal tissue histology was performed at 14 days post-*C*. *parvum* infection. Three-cm segments of ileum were cut in cross section and fixed in 10% zinc-formalin for 48 hours prior to transfer into 70% ethanol. Ileal villus length and crypt depth (≥10 villus:crypt pairs/mouse) were measured in a blinded manner (HH) as previously described using Image J software [23].

Ileal tissues from 4-week-old mice fed either 20% control diet or 2% protein deficient diet for 7 days prior to *C*. *parvum* 10^7^ challenge were harvested on day 4 post-infection. Ileal tissue was cut into 0.5 cm segments and embedded in an optimum cutting temperature (OCT) media-filled cryomold on dry ice. Embedded tissues were stored at -80°C. Frozen sections (5 μm) were fixed in 1% paraformaldehyde in phosphate-buffered saline and immunostained with mouse anti–ZO-1 (Invitrogen), rabbit anti–claudin-2 (Abcam), or mouse anti–occludin (Invitrogen) followed by Alexa Fluor 488– or Alexa Fluor 594–conjugated secondary antibodies (Invitrogen), along with Hoechst 33342 (Invitrogen). Stained sections were mounted in ProLong Gold (Invitrogen) and images were captured using a Coolsnap HQ camera (Roper Scientific) mounted on an Axioplan 2 epifluorescence microscope equipped with a Plan-Neofluar 63× NA 1.3 objective (Zeiss) and ET-sputtered single band filter sets (Chroma Technology) [89]. The microscope was controlled using MetaMorph 7 (Molecular Devices). Exposure times were matched between conditions for each antigen, and all post-acquisition processing was standardized for each antigen. Overlays were created using MetaMorph 7 and subsequently rotated using Adobe Photoshop CS6.

### *Salmonella enterica* Typhi CVD 908-htr vaccination

The *S*. Typhi CVD 908-htr vector transfected with the pSEC10 plasmid expressing ClyA and ClyA-sporozoite surface antigen fusion proteins was generated at the University of Maryland Center for Vaccine Development and prepared at Virginia Commonwealth University (VCU) as previously published [26, 34]. The surface sporozoite antigens Cp15 and CApy were identified using a reverse vaccinology approach and initial immunogenicity studies were performed at Virginia Commonwealth University [90]. The optimized vaccine protocol utilized sequential intranasal prime inoculation with 5x10^9^ live *S*. Typhi at two week intervals followed two weeks later by an intramuscular boost with 20 μg of homologous recombinant Cp15, CApy, or NUS control-peptide mixed 1:1 with alum adjuvant [26]. *S*. Tyhpi aliquots were prepared fresh for each inoculation at VCU and transported to UVa for immediate use. Mice remained on the house vivarium chow throughout the duration of vaccine protocol and for another 51 days prior to transitioning to customized diets. Each mouse was weighed periodically throughout the vaccination and malnutrition protocol. In a separate experiment interval stools were collected for *Salmonella* detection using TaqMan (AgPath-ID OneStep RT-PCR kit (Life Technologies, Cat#4387391) for the *invA* target (5’-3’ sequences: F- TCGGGCAATTCGTTATTGG; R- GATAAACTGGACCACGGTGACA; Probe-FAM-AAGACAACAAAACCCACCGC-MGB) [28] to confirm absence of shedding 24 hours after the first intranasal dose and immediately before the second intranasal dose.

### Re-challenge experiments

Infection with 10^6^ or 10^7^ *Cryptosporidium* oocysts, intranasal 5x10^9^ live *S*. Typhi, or 100 μg intranasal CpG-ODNs (1668 5’TCCATGAGCTTCCTGATGCT’3; Sigma-Aldrich) occurred after 5–7 days of conditioning on the 2% protein diet at 26–28 days of life. The dose of CpG-ODN was determined from prior experience [24] and pilot experiments. *S*. Typhi for these experiments was regrown from frozen aliquots from an initial shipment from VCU using previously published methods [26]. Mice were weighed serially following initial challenge for 20–22 days. On post-challenge day 20–22, mice were re-challenged with 10^7^ *C*. *parvum* oocysts and weighed daily thereafter for 3–10 days. Stools were collected every other day following primary or secondary challenge.

#### DNA extraction and real-time PCR for parasite detection

DNA was extracted from thawed stool stamples using QIAmp DNA stool extraction protocol on the QIAcube (Qiagen) with minor modifications. DNA from tissue samples was extracted from frozen tissue samples using QIAmp DNA tissue extraction protocol on the QIAcube (Qiagen). Qiagen reagents were used for all extractions. The master mix solution and primer sequence targeting the 18S ribosomal subunit utilized are described in detail elsewhere (5’-3’ primers: forward: CTGCGAATGGCTCATTATAACA, reverse: AGGCCAATACCCTACCGTCT) [91]. Quantification of the infection was performed in a Bio-Rad iCycler iQ PCR Detection System by interpolating Ct values of each run with a standard curve of known amounts of *C*. *parvum* ranging from 10^6^ to 10^1^ and transformed into number of organisms per 10 milligrams of stool or tissue sample. The optimized protocol consisted of amplification for 5 minutes at 95°C, followed by 40 cycles of 10 seconds at 95°C and 30 seconds at 60°C, followed by melt curve analysis starting at 60°C with 0.5°C increments. The limit of detection of was set at 10^1^/10 mg stool given that at this Ct value (~36) the specificity of the assay diminished and samples with a Ct value >37 were assigned a ‘zero’ value designating undetectable.

### Immunology assays

#### Antigen-specific serum antibody responses

Antigen-specific serum IgG antibody titers were determined using a customized ELISA in microtiter plates (96 wells) coated with recombinant protein (2 μg/ml rCp15 or rCApy) as previously described [34]. Geometric mean titers were calculated using GraphPad Prism 6. Controls were defined as uninfected, unvaccinated mice on CD-diet and fold-changes were derived relative to this group.

#### Recall assays

Post-stimulation cytokine profiles from splenocytes and mesenteric lymph nodes were determined as previously described [26]. Briefly, cells were harvested from mice at the UVa and transported in RPMI 1640 with 10% FBS and antibiotic-antimycotic to VCU. Specimens from individual mice or pooled were plated and cultured in medium (RPMI 1640 with10% FBS) containing recombinant Cp15 protein (10 mg/mL), rCApy (10 mg/mL), crude *Cryptosporidium parvum* oocyst lysate (10 mg/mL) or in medium alone. Concanavalin A (ConA. 10 μg/mL) was used as a positive control for proliferation. Optimal concentrations of rCp15, rCApy, *C*. *parvum* lysate, and ConA were established in previous studies. After incubation for 3 days at 37°C in an atmosphere containing 5% CO2, multiplex cytokine quantification was performed on 60 μl aliquots of post-stimulation supernatants using the Luminex 100 IS System at the UVa Biomolecular Core facility. Controls were defined as uninfected, unvaccinated mice on CD-diet and fold-changes were derived relative to this group.

#### Flow cytometry

Flow cytometry of lamina propria cells was performed according to our previously published protocols [31, 92]. For isolation of cells from ileum segments, suspensions of small intestinal lamina propria cells were prepared from 4 cm segments of distal small intestine beginning 1 cm from the ileocecal valve. After segments were PBS-flushed and cleaned of gross debris and mucus, they were incubated at 37°C in HBSS buffer containing 50mM EDTA and 1mM DTT for 30 minutes in a shaking incubator at 250 rpm in order to remove epithelial-layer cells. The digested tissue was passed through a 100-μm filter and the filtrate centrifuged as previously described [92]. For lamina propria cell isolations, the tissue pieces were minced and suspended in 10 ml RPMI media with 4% FBS containing 1.2 mg/ml collagenase Type IV, 1.0 mg/ml dispase, and 25–40 U/ml DNase I enzyme solution for 30 minutes at 37°C in a shaking incubator and strained through a 40-μm filter. The resulting pellets were resuspended in 1% BSA-PBS buffer. Fluorophore-conjugated purified mAbs used in flow cytometry were purchased from BD Biosciences (CD8-FITC, CD4-PE-Cy7, NK1.1-PE, CD3ε-BV421, CD45-V500, CD11c-APC-Cy7, and CD103-APC) and Biolegend (B220 [CD45R]-PerCP), and cell surface staining was performed according to the manufacturer’s instructions. All samples were acquired on a CyAn ADP LX analyzer (BD Biosciences and Cytek Development). The leukocyte population was gated based on forward/side scatter and equal events within the gate collected (i.e., 500,000). Cell analysis was performed using FlowJo version 9.3.3 software (Tree Star).

#### Intestinal cytokine secretion

For mucosal cytokine and chemokine responses, 0.5–1.0 cm of ileum were immediately placed in liquid nitrogen at the time of euthanasia and stored at -80°C until use. Protein was collected from ileum lysates, which were made using a lysis buffer containing 50 mM HEPES, 1% Triton X-100, and Halt protease inhibitor on ice and homogenized in Zirconia beads (Biospec) using a Mini-Beadbeater (Biospec) for 60 seconds. Clarified supernatants were stored at -80°C. Multiplex protein quantification was performed using Luminex 100 IS System at the University of Virginia Biomolecular Core facility. Cytokine and chemokine levels were normalized to total lysate protein as determined by bicinchoninic assay (BCA) (Thermoscientific) at 562nm absorbance (Biotek ELISA plate reader) after 30 minutes incubation of sample with reagent at room temperature.

#### IL17 gene expression

Total cellular RNA was obtained from each intestinal tissue using an RNeasy kit, and cDNA was synthesized from 1 μg RNA using iScript. For quantitative PCR analyses of cytokine mRNA abundance, the cDNA was diluted 1: 8 and 4 μl of this dilution was used for each PCR. Reagents from the real-time PCR kit containing SYBR Green were used for quantitative PCR assays. The primer sequences used were as follows: *β-actin*, sense 5′-CCACCATGTACCCAGGCATT-3′ and antisense 5′-CGGACTCATCGTACTCCTGC-3′*; IL-17*, sense 5′-AGGGAGCCTGAGAGCTGCCC-3′ and antisense 5′-AATCGAGGCCACGCAGGTGC-3′; The PCR conditions were: 95°C for 13 min, followed by 40 cycles of 95°C for 30 s, 58°C for 30 s and 72°C for 30 s, followed by melt-curve analysis. Data were analysed and are presented based on the relative expression method as previously described [30].

### Statistical analysis

Data analyses were performed with GraphPad Prism 6 software (GraphPad Software). All statistical analyses were done with the use of analysis of variance, Student *t* tests, and Bonferroni or Tukey post hoc analysis where applicable. Differences were considered significant at *P* <0.05. Data are represented as means ± standard errors of the mean unless otherwise specified.

## Supporting Information

S1 Fig Effect of select nutrient deficiency (zinc or protein) on cryptosporidiosis. (A) Growth of mice infected with *C*. *parvum* on protein deficient (PD) compared with isolated zinc deficient diet (ZD) (n = 4/group). **P<0.00 and ****P<0.0001 for *C*. *parvum*pd vs. PBSpd; ##*P*<0.00 and ####*P*<0.0001 for *C*. *parvum*^zd^ vs. PBS^pd^; ^^^^*P*<0.0001 for *C*. *parvum*^zd^ vs. *C*. *parvum*^pd^. (B) Fecal parasite shedding per 10 mgfecesasdetermined by RT-PCR. (C) Growth of control diet (CD) and protein deficient (PD) diet-fed mice beginning 12 days prior to *C*. *parvum* infection. Mice were challenged on experimental day 0. * *P*<0.05 and ***P<0.0001 for *C*. *parvum*pd vs PBS^pd^, ^*P*<0.05 for *C*. *parvum*^pd^ vs *C*. *parvum*, 2-way ANOVA, Bonferroni post-test anlaysis. (D) Fecal parasite shedding per 10 mg feces as determined by RT-PCR. The break in the y-axis of the graph represents the assay limit of detection, and ‘0’ indicates no detection. *P<0.05, Student’s *t*-test. (E) Ileal intestinal morphometry measured as villus length, crypt depth, andvillus:cryptratios. Data are representative of tissues at 13–15 days post-infection (n = 10 villus/crypt pairs per animal). *P<0.05, ** *P*<0.01, ***P<0.001 One-Way ANOVA, Tukey post-test analysis.
(PDF)

S2 Fig Effect of *C*. *parvum* 10^6^ priming on ileum CD45+ cell populations at three days post-challenge. Numbers of (A) dendritic cells (CD11c^+^), (B) B-cells (B220^+^), (C) T-cells (CD3ε^+^), (D) CD4^+^ and (E) CD8^+^ cells as % relative to CD45+ events.
(PDF)

S3 Fig Mouse growth during vaccination with *Cryptosporidium* sporozoite protein expressing *S*. Typhi in nourished and protein malnourished mice. Growth during the vaccination and malnutrition protocol, prior to Cryptosporidium challenge. Mice were randomized into intervention groups (PBS- sham, S. Typhi, S. Typhi^Cp15^, or S. Typhi^CApy^), and then to be challenged with either C. parvum or PBS control. (A) Depicts growth of each group of mice during the vaccination period. Inset bar graph is weights on day 107 when mice were transitioned to either control or protein-deficient diet (pd). (B) Absolute weight of mice after 12 days on either the control or protein-deficient (pd) diets. Top: Mice are grouped according to whether they were allocated to be challenged with either *C*. *parvum* or PBS. Bottom: Distribution of weights for all mice according to intervention arm. (C,D) Effects of 12 days of control (C) or protein-deficient diet (pd) (D) in each individual group shown as % of initial weight. For all figures, **P<0.001 and *P<0.05. (E) Serum IgG anti-Cp15 or anti-CApy to homologous vaccinogen for uninfected S. Typhi^Cp15^ or S. Typhi^CApy^ vaccinated mice compared with unvaccinated controls (pooled either *S*. Typhi or PBS) at 75–77 days after vaccination. (left) *P<0.05 for S. Typhi^Cp15^ vs unvaccinated in CD-fed mice; #*P*<0.05 S. Typhi^Cp15^ vs unvaccinated in PD-fed mice. (right) mice grouped by vaccine group only, regardless of diet. (F) Cytokine secretion after stimulation with homologous vaccinogens in pooled mesenteric lymph nodes from uninfected vaccinated mice compared with uninfected unvaccinated PBS controls. Control diet (CD) (left) and PD-fed (right) mice as indicated.
(PDF)

S4 Fig Effect of any remote *S*. Typhi exposure on *C*. *parvum* challenge, ileal IL17A expression, and *C*. *parvum* challenge outcomes in IL17RA^-/-^ mice. (A) Control-diet fed growth curves. (**B**) Protein-deficient (PD)-fed growth curves. Significant values are indicated as * for *P*<0.05 (red = all uninfected vs. PBS infected; green = All *S*. Typhi infected vs. PBS infected; black = all uninfected vs. All *S*. Typhi infected); ** for *P*<0.01 *** for *P*<0.001 **** for *P*<0.0001, respectively. (**C**) Post *C*. *parvum* infection shedding by RT-PCR in serial fecal pellets in pd-fed animals aggregated as All *S*. Typhi infection or PBS-only. Significant values are indicated as * for *P*<0.05. (D) *S*. Typhi RT-PCR in feces of mice prior to exposure, 24 hours after intranasal exposure, and at 2 weeks post-exposure (n = 8/group). (E) IL17 mRNA expression normalized to house keeping gene measured in the ileum at seven weeks after intranasal exposure to the *S*. Typhi vector expressed as fold change relative to intranasal PBS exposure (n = 4–7 per group). (F) Growth (left) and *C*. *parvum* shedding (right) in wild type C57Bl/6 and IL17RA KO mice through three days after 10^7^ *C*. *parvum* challenge (n = 3-4/group).
(PDF)

S5 Fig Impact of various routes and combinations of non-specific innate immune adjuvants (CpG-ODN 1668 and *S*. Typhi vector) on experimental cryptosporidiosis. 8 week old CD-fed C57Bl/6 females were administered either CpG-ODN 1668 (intranasal = IN), the *S*. Typhi vector, or PBS-sham intranasally, and again at 10 weeks of life. All mice also received either intramuscular alum or intramuscular alum+NUS at 12 weeks of life. Mice were transitioned to PD diet at 13 weeks of life and challenged with *C*. *parvum* 10^7^ 2 weeks later. CpG-ODN intraperitoneal (IP) or PBS IP was given to each of two previously sham-only treated groups beginning 3 days prior, the day of *C*. *parvum*, and 3 days after *C*. *parvum* challenge. A) Growth as percentage of initial weight on the day of *C*. *parvum* challenge. *P*<0.05 for CpG-ODN IN vs PBS-any sham. B) The “PBS-any sham group, n = 12” represents 3 separate groups (n = 4 each) consisting of either PBS+alum, PBS+alum+NUS, or a sham intraperitoneal CpG-ODN controls. Growth was similar in all sham groups. C) Post-challenge shedding by 18S RT-PCR. *P*<0.05 for indicated groups. D) Comparison of CpG with *S*. Typhi or each alone for growth and E) parasite shedding. For D,E) There were no significant differences between any of the groups.
(PDF)

S6 Fig **Growth after inoculation with either PBS, *S*. Typhi *908htr* intranasal, CpG-ODN 1668 intranasal, or *C*. *parvum* 10^6^ orogastric gavage through 22 days as absolute weight in grams (A) and percentage of initial weight (B).** (C) Parasite burden as Log10 per 10 mg fecal pellet after primary challenge with *C*. *parvum* 10^6^. (D) Growth as percentage of initial weight beginning on experimental day 23 (post re-challenge day 0) for indicated groups. The groups labeled PBS-PBS, *S*. Typhi-PBS, and CpG-PBS are aggregated as “All uninfected” in Fig 8A and 8B. (E) Ileal CCL, IL12p40, and IFNγ at 3 days after *C*. *parvum* 10^7^ challenge in mice previously primed with either *C*. *parvum* or Δ*C*. *parvum* both at 10^6^ inoculum compared with PBS controls. *P<0.05, ***P<0.001 as indicated (n = 5/group).
(PDF)

S7 Fig Gating strategy for mucosal T-cell subsets. (PDF)
