# Supplementary figures and images for: Cryptosporidium Priming Is More Effective than Vaccine for Protection against Cryptosporidiosis in a Murine Protein Malnutrition Model
Source: PLoS Negl Trop Dis. 2016 Jul 28;10(7):e0004820. doi: 10.1371/journal.pntd.0004820 (PMC4965189; doi:10.1371/journal.pntd.0004820)

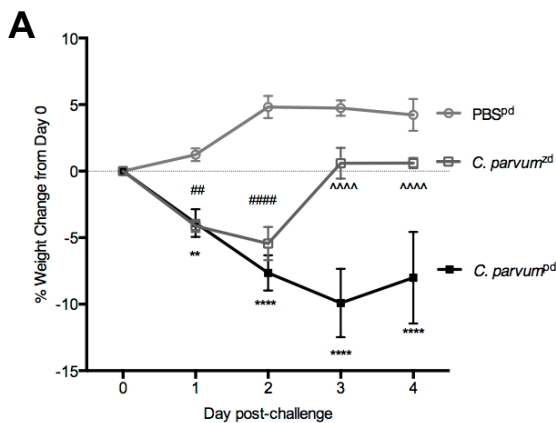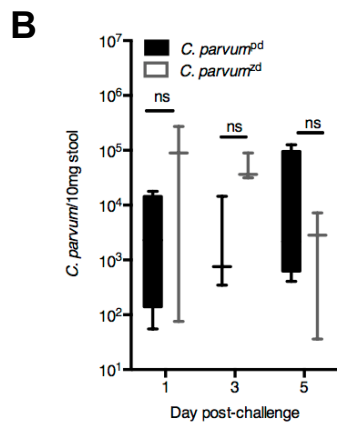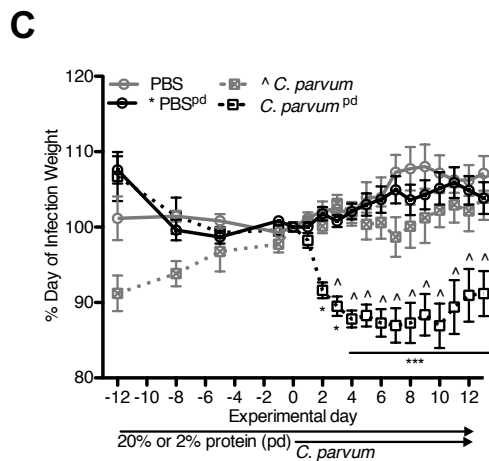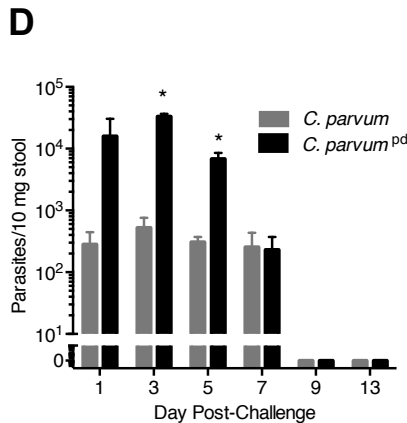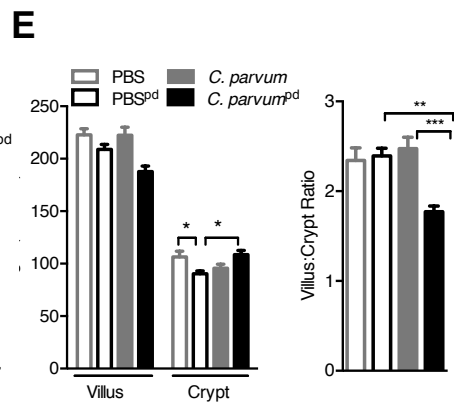

Supplement: S1 Fig — (A) Growth of mice infected with C. parvum on protein deficient (PD) compared with isolated zinc deficient diet (ZD) (n = 4/group). **P<0.00 and ****P<0.0001 for C. parvumpd vs. PBSpd; ##P<0.00 and ####P<0.0001 for C. parvumzd vs. PBSpd; ^^^^P<0.0001 for C. parvumzd vs. C. parvumpd. (B) Fecal parasite shedding per 10 mgfecesasdetermined by RT-PCR. (C) Growth of control diet (CD) and protein deficient (PD) diet-fed mice beginning 12 days prior to C. parvum infection. Mice were challenged on experimental day 0. * P<0.05 and ***P<0.0001 for C. parvumpd vs PBSpd, ^P<0.05 for C. parvumpd vs C. parvum, 2-way ANOVA, Bonferroni post-test anlaysis. (D) Fecal parasite shedding per 10 mg feces as determined by RT-PCR. The break in the y-axis of the graph represents the assay limit of detection, and ‘0’ indicates no detection. *P<0.05, Student’s t-test. (E) Ileal intestinal morphometry measured as villus length, crypt depth, andvillus:cryptratios. Data are representative of tissues at 13–15 days post-infection (n = 10 villus/crypt pairs per animal). *P<0.05, ** P<0.01, ***P<0.001 One-Way ANOVA, Tukey post-test analysis. (PDF) [file pntd.0004820.s001.pdf]

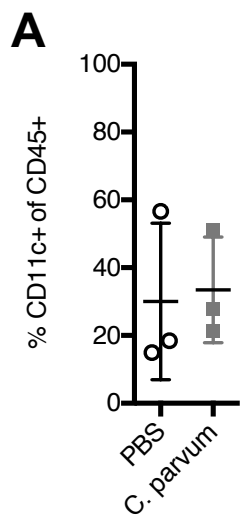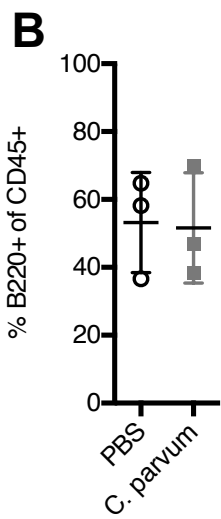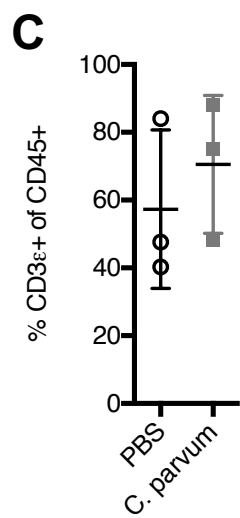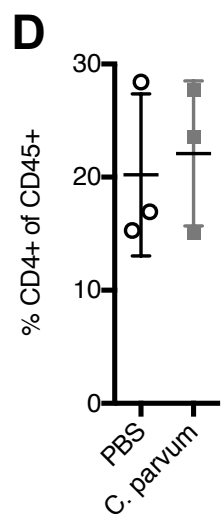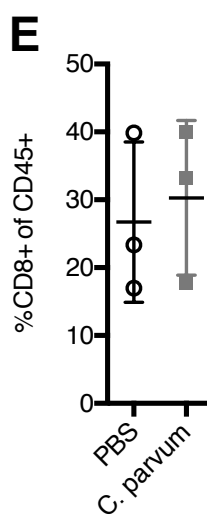

Supplement: S2 Fig — Numbers of (A) dendritic cells (CD11c+), (B) B-cells (B220+), (C) T-cells (CD3ε+), (D) CD4+ and (E) CD8+ cells as % relative to CD45+ events. (PDF) [file pntd.0004820.s002.pdf]

**A**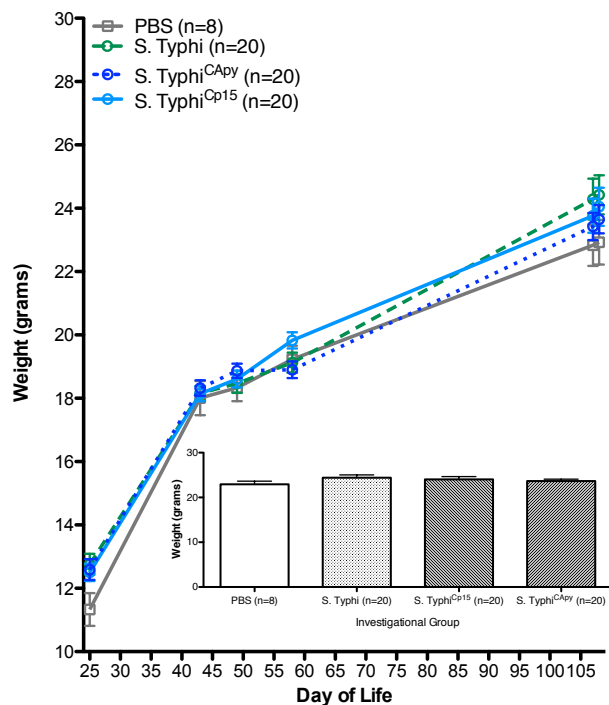**B**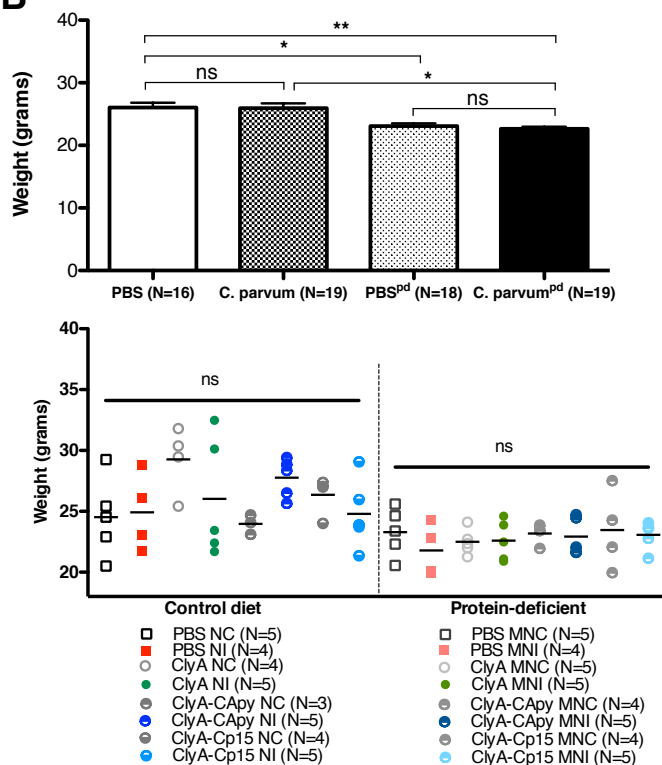**C**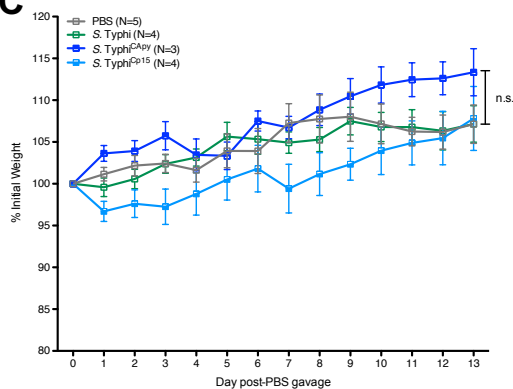**D**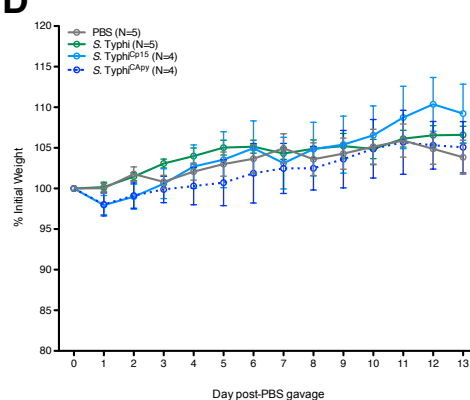**E**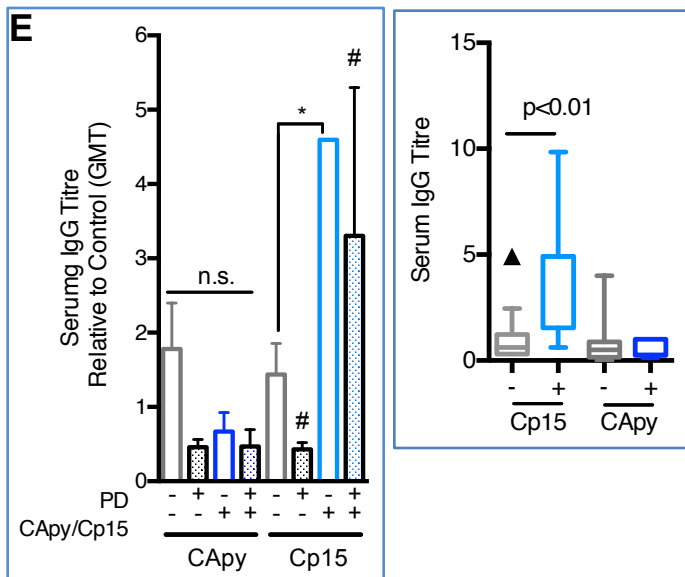**F**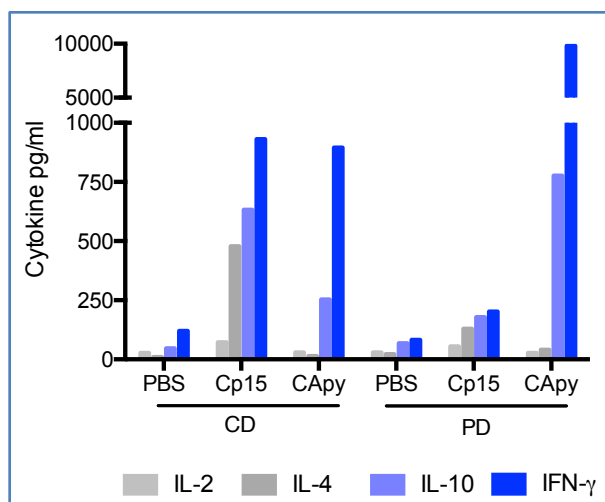

Supplement: S3 Fig — Growth during the vaccination and malnutrition protocol, prior to Cryptosporidium challenge. Mice were randomized into intervention groups (PBS- sham, S. Typhi, S. TyphiCp15, or S. TyphiCApy), and then to be challenged with either C. parvum or PBS control. (A) Depicts growth of each group of mice during the vaccination period. Inset bar graph is weights on day 107 when mice were transitioned to either control or protein-deficient diet (pd). (B) Absolute weight of mice after 12 days on either the control or protein-deficient (pd) diets. Top: Mice are grouped according to whether they were allocated to be challenged with either C. parvum or PBS. Bottom: Distribution of weights for all mice according to intervention arm. (C,D) Effects of 12 days of control (C) or protein-deficient diet (pd) (D) in each individual group shown as % of initial weight. For all figures, **P<0.001 and *P<0.05. (E) Serum IgG anti-Cp15 or anti-CApy to homologous vaccinogen for uninfected S. TyphiCp15 or S. TyphiCApy vaccinated mice compared with unvaccinated controls (pooled either S. Typhi or PBS) at 75–77 days after vaccination. (left) *P<0.05 for S. TyphiCp15 vs unvaccinated in CD-fed mice; #P<0.05 S. TyphiCp15 vs unvaccinated in PD-fed mice. (right) mice grouped by vaccine group only, regardless of diet. (F) Cytokine secretion after stimulation with homologous vaccinogens in pooled mesenteric lymph nodes from uninfected vaccinated mice compared with uninfected unvaccinated PBS controls. Control diet (CD) (left) and PD-fed (right) mice as indicated. (PDF) [file pntd.0004820.s003.pdf]

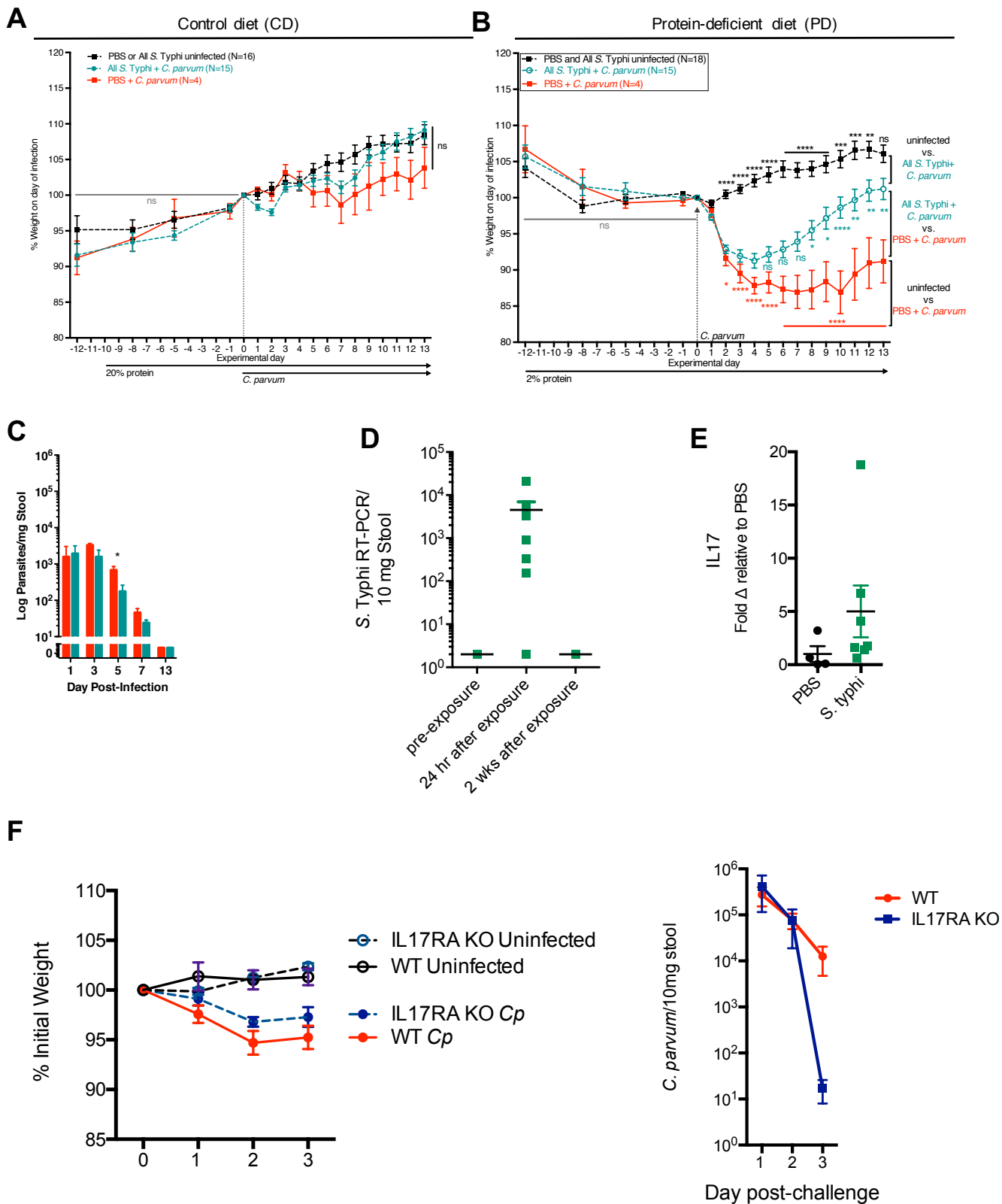

Supplement: S4 Fig — (A) Control-diet fed growth curves. (B) Protein-deficient (PD)-fed growth curves. Significant values are indicated as * for P<0.05 (red = all uninfected vs. PBS infected; green = All S. Typhi infected vs. PBS infected; black = all uninfected vs. All S. Typhi infected); ** for P<0.01 *** for P<0.001 **** for P<0.0001, respectively. (C) Post C. parvum infection shedding by RT-PCR in serial fecal pellets in pd-fed animals aggregated as All S. Typhi infection or PBS-only. Significant values are indicated as * for P<0.05. (D) S. Typhi RT-PCR in feces of mice prior to exposure, 24 hours after intranasal exposure, and at 2 weeks post-exposure (n = 8/group). (E) IL17 mRNA expression normalized to house keeping gene measured in the ileum at seven weeks after intranasal exposure to the S. Typhi vector expressed as fold change relative to intranasal PBS exposure (n = 4–7 per group). (F) Growth (left) and C. parvum shedding (right) in wild type C57Bl/6 and IL17RA KO mice through three days after 107 C. parvum challenge (n = 3-4/group). (PDF) [file pntd.0004820.s004.pdf]

**A**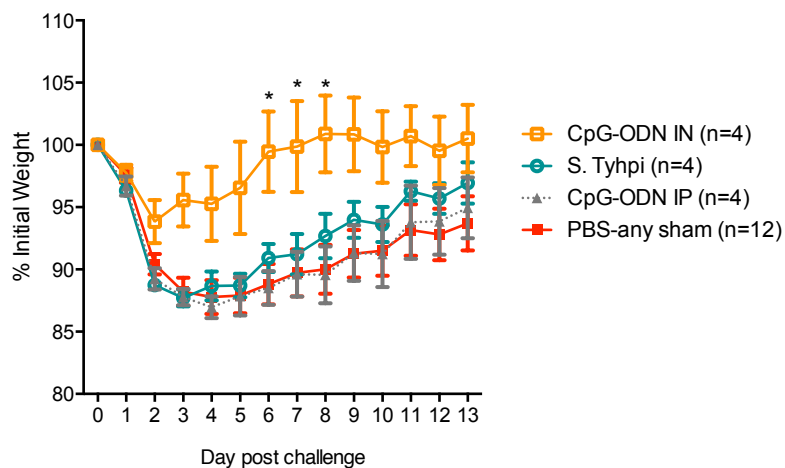**B**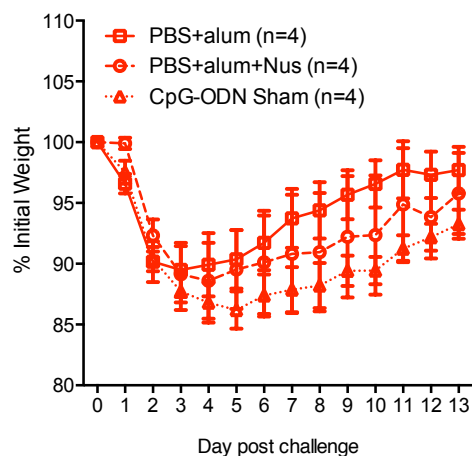**C**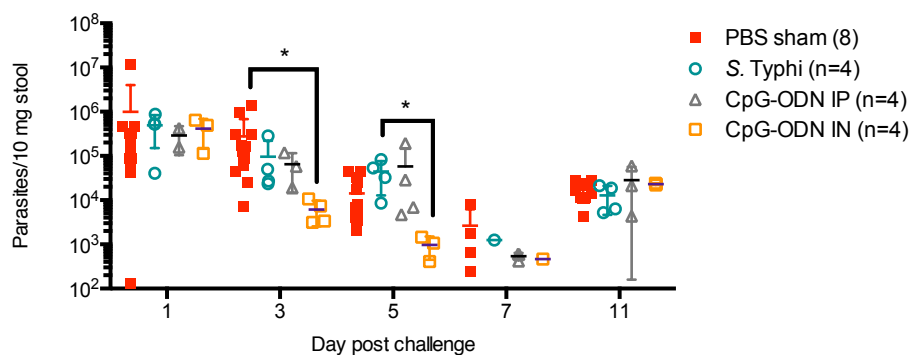**D**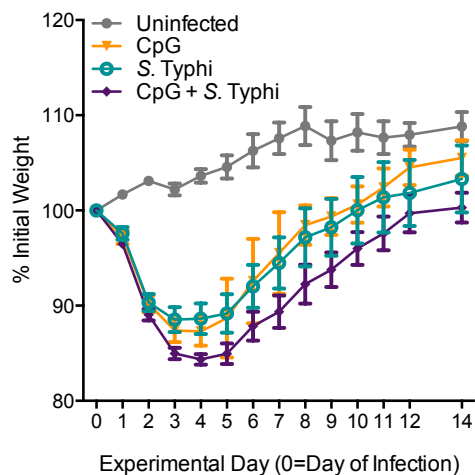**E**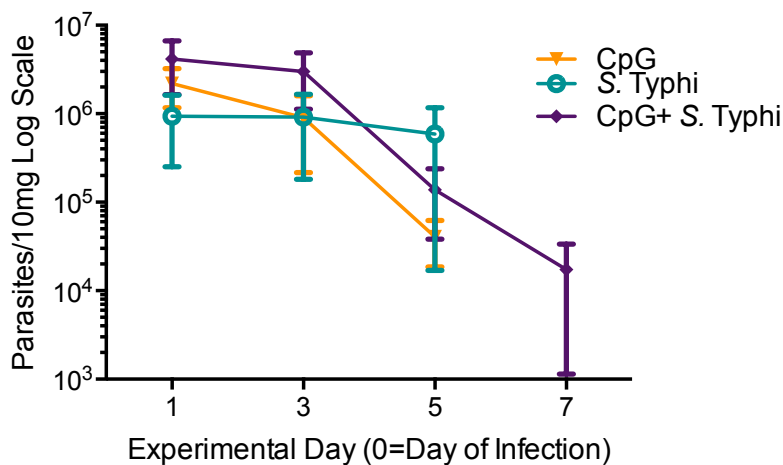

Supplement: S5 Fig — 8 week old CD-fed C57Bl/6 females were administered either CpG-ODN 1668 (intranasal = IN), the S. Typhi vector, or PBS-sham intranasally, and again at 10 weeks of life. All mice also received either intramuscular alum or intramuscular alum+NUS at 12 weeks of life. Mice were transitioned to PD diet at 13 weeks of life and challenged with C. parvum 107 2 weeks later. CpG-ODN intraperitoneal (IP) or PBS IP was given to each of two previously sham-only treated groups beginning 3 days prior, the day of C. parvum, and 3 days after C. parvum challenge. A) Growth as percentage of initial weight on the day of C. parvum challenge. P<0.05 for CpG-ODN IN vs PBS-any sham. B) The “PBS-any sham group, n = 12” represents 3 separate groups (n = 4 each) consisting of either PBS+alum, PBS+alum+NUS, or a sham intraperitoneal CpG-ODN controls. Growth was similar in all sham groups. C) Post-challenge shedding by 18S RT-PCR. P<0.05 for indicated groups. D) Comparison of CpG with S. Typhi or each alone for growth and E) parasite shedding. For D,E) There were no significant differences between any of the groups. (PDF) [file pntd.0004820.s005.pdf]

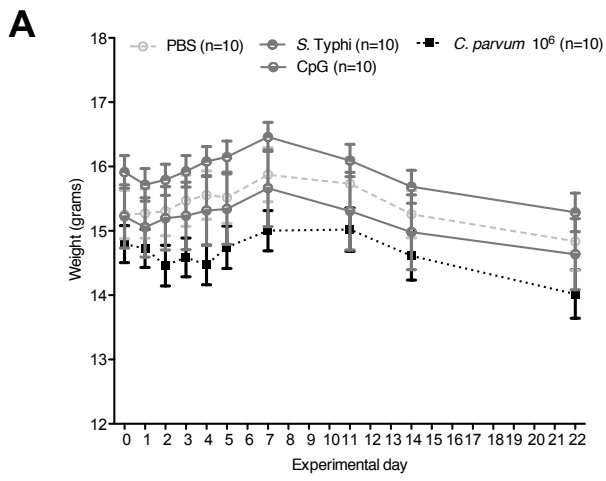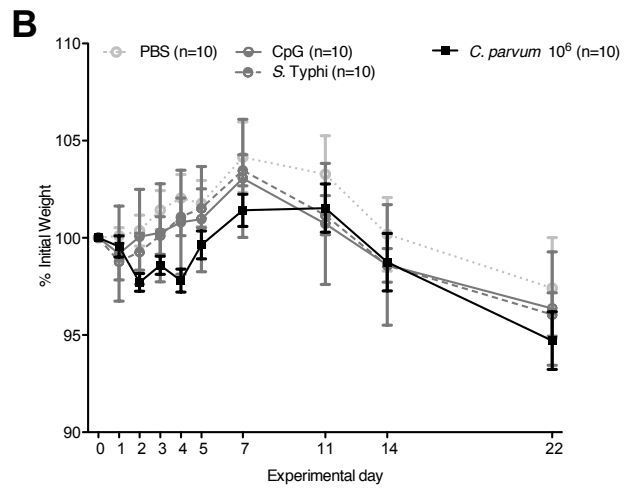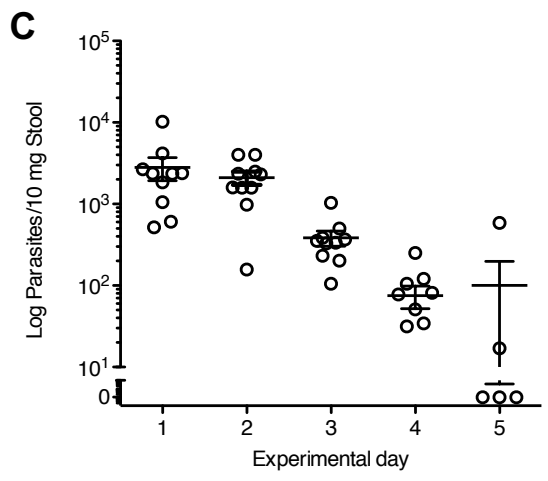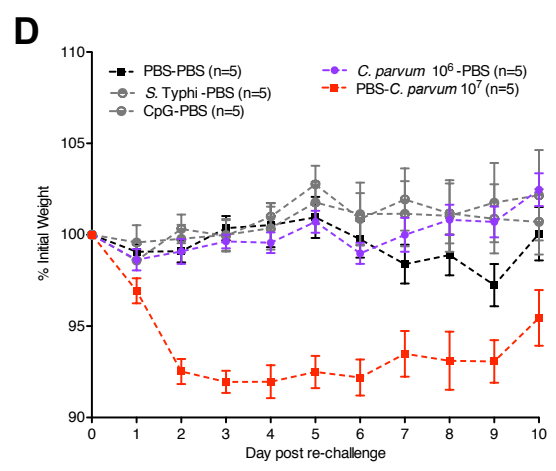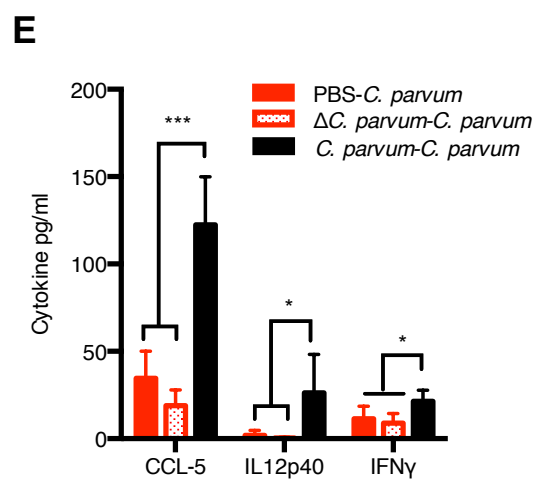

Supplement: S6 Fig — Growth after inoculation with either PBS, S. Typhi 908htr intranasal, CpG-ODN 1668 intranasal, or C. parvum 106 orogastric gavage through 22 days as absolute weight in grams (A) and percentage of initial weight (B). (C) Parasite burden as Log10 per 10 mg fecal pellet after primary challenge with C. parvum 106. (D) Growth as percentage of initial weight beginning on experimental day 23 (post re-challenge day 0) for indicated groups. The groups labeled PBS-PBS, S. Typhi-PBS, and CpG-PBS are aggregated as “All uninfected” in Fig 8A and 8B. (E) Ileal CCL, IL12p40, and IFNγ at 3 days after C. parvum 107 challenge in mice previously primed with either C. parvum or ΔC. parvum both at 106 inoculum compared with PBS controls. *P<0.05, ***P<0.001 as indicated (n = 5/group). (PDF) [file pntd.0004820.s006.pdf]

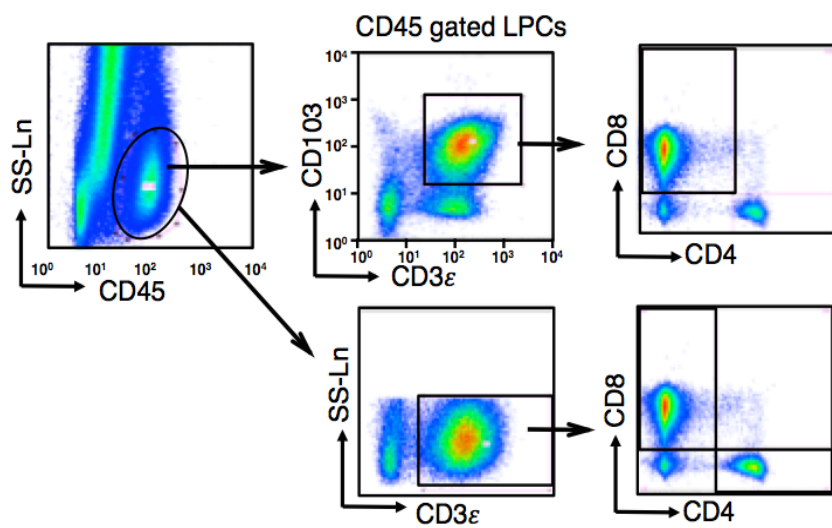

Supplement: S7 Fig — (PDF) [file pntd.0004820.s007.pdf]
